# Targeting glycerophospholipid biosynthesis overcomes chemoresistance driven by SLFN11 loss in Ewing sarcoma

**DOI:** 10.1038/s41419-026-08432-7

**Published:** 2026-01-31

**Authors:** Kasturee Chakraborty, Ritambhar Burman, Saharsh Satheesh, Matthew Kieffer, Chandni Karuhatty, Zuo-Fei Yuan, Haiyan Tan, Ankhbayar Lkhagva, Anthony A. High, Xusheng Wang, Alaa Refaat, Nathaniel R. Twarog, Weixing Zhang, Yaxu Wang, Yiping Fan, Qian Li, M. Madan Babu, Anang A. Shelat, Elizabeth Stewart, Michael A. Dyer, Puneet Bagga

**Affiliations:** 1 Department of Radiology, St. Jude Children’s Research Hospital, Memphis, TN USA; 2 Department of Developmental Neurobiology, St. Jude Children’s Research Hospital, Memphis, TN USA; 3 Center for Proteomics and Metabolomics, St. Jude Children’s Research Hospital, Memphis, TN USA; 4 Department of Neurology, University of Tennessee Health Science Center, Memphis, TN USA; 5 Department of Genetics, Genomics and Informatics, University of Tennessee Health Science Center, Memphis, TN USA; 6 Department of Chemical Biology and Therapeutics, St. Jude Children’s Research Hospital, Memphis, TN USA; 7 Department of Structural Biology, St. Jude Children’s Research Hospital, Memphis, TN USA; 8 Center of Excellence for Data-Driven Discovery, Department of Structural Biology, St. Jude Children’s Research Hospital, Memphis, TN USA; 9 Center for Applied Bioinformatics, St. Jude Children’s Research Hospital, Memphis, TN USA; 10 Department of Biostatistics, St. Jude Children’s Research Hospital, Memphis, TN USA; 11 Department of Oncology, St. Jude Children’s Research Hospital, Memphis, TN USA

**Keywords:** Cancer metabolism, Metabolomics

## Abstract

Ewing sarcoma (EWS) is a highly aggressive pediatric malignancy characterized by elevated expression of *SLFN11*, which impairs DNA repair by binding to and functionally inhibiting DNA repair complexes, thereby enhancing susceptibility to genotoxic therapies. However, relapse remains a major clinical challenge and is often accompanied by the emergence of therapeutic resistance linked to reduced *SLFN11* expression. We hypothesized that *SLFN11*-deficient tumors undergo adaptive metabolic reprogramming to overcome chemosensitivity. Here, we leverage transcriptomic and metabolomic profiling in patient-derived EWS models to demonstrate that *SLFN11* loss drives downregulated mitochondrial glycerol-3-phosphate dehydrogenase (*GPD2*) expression, higher accumulation of glycerol-3-phosphate, fatty acid unsaturation, and enhanced glycerophospholipid (GPL) biosynthesis. Subsequently, targeting GPL biosynthesis (FSG67) restored DNA-damaging agent (SN-38) sensitivity in *SLFN11*-deficient EWS model, revealing a potential metabolic vulnerability to overcome chemoresistance. Furthermore, *SLFN11* knockout tumors exhibited an elevated phosphocholine/glycerophosphocholine ratio, offering a potential non-invasive diagnostic biomarker.

## Introduction

Ewing sarcoma (EWS) is a highly aggressive pediatric cancer that primarily affects bone and soft tissues [1]. Current first-line therapy relies on intensive multimodal regimens combining surgery or radiation [2] with DNA damaging agents (DDA) such as topoisomerase inhibitors [3–5] and alkylating agents [4–7], such as interval-compressed vincristine, doxorubicin, and cyclophosphamide alternating with ifosfamide and etoposide (VDC/IE) or vincristine, ifosfamide, doxorubicin, and etoposide (VIDE) [5]. These approaches are effective in localized disease but offer limited benefit following relapse [2, 4]. Approximately 30–40% of EWS patients develop recurrent tumors showing metastatic progression [8], and salvage regimens including topotecan-cyclophosphamide, irinotecan-temozolomide, gemcitabine-docetaxel, and high-dose ifosfamide yield only transient responses [5], with 3- and 5-year event-free survival rates of 13% and 12%, respectively [5, 9], underscoring the severe impact of chemoresistance. Emerging strategies involving tyrosine kinase inhibitors (cabozantinib, regorafenib, lenvatinib), PARP and EZH2 inhibitors, and immunotherapies such as PD-1 blockade or CAR-T cells have shown modest or inconsistent efficacy [5].

The chemosensitivity in EWS is largely driven by high expression of *SLFN11* [10, 11], a DNA damage response protein that blocks DNA repair and induces cell cycle arrest under therapeutic stress, ultimately leading to cell death [12–15]. Poor therapeutic response in relapsed EWS is associated with heterogeneous *SLFN11* expression during disease progression [15]. Approximately 10% of EWS tumors lack *SLFN11* expression at diagnosis or lose the expression after relapse or over the course of treatment [15]. Despite initial sensitivity in localized EWS, post-relapse outcomes are poor as therapeutic options at relapse remain limited, emphasizing the need for effective strategies in *SLFN11*-deficient EWS [15]. Although *SLFN11* is established as a biomarker of treatment response [11–14, 16, 17], its downstream effects remain poorly understood. Upstream regulation of *SLFN11* indicates that enhanced EZH2 activity during chemoresistance silences *SLFN11* via H3K27 trimethylation [18]. Conversely, EZH2 inhibition restores *SLFN11* expression and re-sensitizes small cell lung cancer to DNA damage [18]. However, to our knowledge no studies have identified targetable vulnerabilities that arise from *SLFN11* loss.

Adaptive metabolic reprogramming is widespread in drug-resistant tumors [19–21], suggesting that *SLFN11* loss may similarly drive survival-promoting metabolic shifts in EWS. To explore this hypothesis, we performed integrated transcriptomic and metabolomic profiling in patient-derived *SLFN11*-knockout models. We observed consistent downregulation of mitochondrial glycerol-3-phosphate dehydrogenase 2 (*GPD2*), leading to glycerol-3-phosphate (G3P) accumulation, enhanced unsaturated fatty acid and glycerophospholipid (GPL) synthesis. In vivo choline metabolism was also found to be rewired, with an elevated phosphocholine to glycerophosphocholine ratio, which may serve as a potential biomarker of this phenotype. Targeting this pathway with a GPL biosynthesis inhibitor (FSG67) [22] restored sensitivity to the DDA (SN-38) in *SLFN11*-knockout cells. Together, these findings highlight GPL biosynthesis as a critical metabolic vulnerability in *SLFN11*-associated chemoresistance and underscore its potential as a therapeutic target in resistant EWS.

## Materials and methods

### mRNA expression profiling

mRNA expression profile for *SLFN11* was analyzed across multiple cancer types using the Cancer Cell Line Encyclopedia (CCLE) database [23]. The analysis included cell lines representing EWS, small cell lung cancer, ovarian cancer, prostate cancer, gastric cancer, breast cancer, colorectal cancer, acute myeloid leukemia, neuroblastoma, renal cell carcinoma, glioblastoma, and hepatocellular carcinoma.

### DepMap-gene dependency analysis

CRISPR-based gene dependency data were obtained from the DepMap database (https://depmap.org/portal) (DepMap version: 24Q4), which was normalized using the Chronos algorithm. Dependency scores represent the likelihood of a gene being essential for cell survival, with lower scores indicating higher dependency. Genes with dependency scores below threshold, [e.g., −0.5] were considered essential. A *t*-test was used to compare the dependency score of *SLFN11* across each cancer lineage or primary disease type against all the cell lines. *p*-values were adjusted using the Benjamini-Hochberg method for false discovery rate (FDR) correction.

### Kaplan-Meier survival analysis protocol for gene expression data

Kaplan Meier survival analysis was performed by categorizing patients into high- and low-expression groups using the median value of *SLFN11* expression as the cutoff. This method iteratively applies ascending gene expression values as thresholds to divide the cohort into two groups and evaluates the *p*-value at each step using a log-rank test. The optimal cutoff value was determined and used to generate the corresponding Kaplan-Meier survival curves. The analysis was conducted on the R2: Genomics Analysis and Visualization Platform (http://r2.amc.nl) [24] using survival and gene expression data from the GSE17679 dataset [25].

### Cell lines and culture

The cells used in this study were kindly provided by Dr. Elizabeth Stewart of St. Jude Children’s Research Hospital. Both WT and *SLFN11*^−/−^ cell lines from a total of five EWS cell lines, ES-8, SK ES-1, EW-8, RD ES-1, and CADO ES-1 were utilized in this study (Table S1). ES-8 and EW-8 cell lines were cultured in DMEM without glutamine (Gibco) with 10% fetal bovine serum (FBS) (Gibco) and 1% penicillin-streptomycin-glutamine (PSG) (Gibco) and maintained at 37 °C in a humidified incubator with 5% CO_2_. SK ES-1 cells were cultured in McCoy’s 5A medium (Gibco) supplemented with 15% FBS and 1% Pen-strep, CADO ES-1 cells were cultured in RPMI 1640 (Gibco), supplemented with 20% FBS and 1% PSG, and RD ES-1 cells were cultured in RPMI 1640, supplemented with 15% FBS and 1% PSG. All cell lines were authenticated by STR profiling and tested to be *Mycoplasma*-free.

### RNA extraction, sequencing, and data analysis

RNA was extracted from in vitro samples using the RNeasy Mini Kit (QIAGEN, Cat. No. 74104). Expression profiles were generated from biological triplicates, as well as biological duplicates of all cell lines. RNA sample quality was assessed using the Agilent 2200 TapeStation System, and data analysis was performed with TapeStation Analysis Software 5.1. RNA-Seq libraries were prepared using the TruSeq Total RNA protocol and sequenced in pools of 5–7 samples per lane on a V3 flow cell (HiSeq 2000/2500). Where necessary, additional sequencing (“top-off”) was performed in rapid mode on the HiSeq 2500 to ensure data analysis was completed within a clinically appropriate time frame [26]. The RNA seq reads were mapped to mouse genome (gencode M22) using STAR2.7 [27]. After mapping, the gene count matrix for each sample was generated by RSEM [28]. The differentially expressed genes were identified using limma package [29] with count matrix as input. The cutoff for differential gene is log_2_ fold change >0.5 and FDR-adjusted *p* ≤ 0.05. Heatmap and volcano plots were generated using in-house scripts.

### Immunoblotting

The following antibodies were used: SLFN11 (Sigma: HPA023030), GPD2 (Proteintech: 17219-1-AP), β-actin (Cell Signaling Technology: 4970), and secondary antibody (Cell Signaling Technology: 7074P2). Cells were lysed using 1X-lysis buffer (Cell Signaling Technology: 9803), vortexed, and centrifuged at 4 °C for 15 min. The supernatant was collected, and protein concentration was measured using the BCA protein quantification assay (Thermo: 23228). Proteins were separated by SDS-PAGE and transferred to PVDF membranes (Merck: ISEQ00005). Membranes were blocked with 5% skimmed milk or BSA for 1 h at room temperature. After blocking, the PVDF membrane was incubated with primary antibodies at 4°C overnight on a rocker. Secondary antibodies were diluted in Tris-buffered saline with Tween-20 (TBST) and added to the membranes, followed by incubation for 2 h at room temperature. Protein bands were visualized using the ChemiDocTM Touch Imaging System. ImageJ software was used for the gray-level analysis.

### Correlation analysis of gene expression

Transcriptomic data were obtained from the Ewing Sarcoma Cell Line Atlas (ESCLA) [24] and the inflammatory gene profiling dataset of the Ewing sarcoma family of tumors [25]. Batch effects due to treatment conditions, cell lines, or diagnostic subtypes were removed using the limma package (version 3.60.4) [29]. Pearson correlation coefficients were calculated on log2-normalized expression values, and the significance of correlations was tested using the cor.test function in R.

### Metabolite extraction

One million cells were seeded in each well of 6-well plates and grown overnight. The cells were washed twice with ice-cold 1X phosphate-buffered saline (PBS), followed by 500 µL of freezing 80% acetonitrile (LC-MS grade, Life Technologies Corp.). The cells were harvested by scrapping into 1.5 mL tubes and lysed in the presence of glass beads by Bullet Blender (Next Advance) at 4 °C until the samples were homogenized. The lysate was centrifuged at 21,000 × *g* for 5 min and metabolite containing supernatant was split into two aliquots and dried by speedvac.

### Untargeted metabolome profiling by LC-MS/MS

One aliquot of metabolite sample was resuspended in 1% acetonitrile plus 0.1% trifluoroacetic acid (100 µL/million cells), 50 μL of the sample was desalted by Ultra-C18 Micro spin columns (Harvard apparatus) and eluted by 125 µL of 80% acetonitrile plus 0.1% trifluoroacetic acid followed by speedvac drying. The sample was then resuspended in 30 μL of 5% formic acid, and 2 μL was analyzed by acidic pH reverse phase LC-MS/MS with a self-packed column (75 μm × 15 cm with 1.9 µm C18 resin from Dr. Maisch) coupled with a Q Exactive HF Orbitrap MS (Thermo Fisher) in positive ion mode. Metabolites were eluted within a 50 min gradient (mobile phase A: 0.2% formic acid in H_2_O; mobile phase B: 0.2% formic acid in acetonitrile; flow rate: 0.25 μL/min). Another aliquot of metabolite sample was resuspended in a solvent containing 45% isopropanol, 5% acetonitrile and 50% H_2_O (20 μL/million cells) and 3 µL was analyzed by a ZIC-HILIC column (150 × 2.1 mm, EMD Millipore) coupled with a Q Exactive HF Orbitrap MS (Thermo Fisher) in negative ion mode. Metabolites were eluted within a 45 min gradient (mobile phase A: 10 mM ammonium acetate in 90% acetonitrile, pH = 8; mobile phase B: 10 mM ammonium acetate in 100% H2O, pH = 8; flow rate: 0.1 mL/min). The mass spectrometry methods for both metabolomics analyses were set up with the following parameters: one MS1 scan (120,000 resolution, 100–1000 m/z, 3 × 10^6^ AGC and 50 ms maximal ion time) followed by 20 data-dependent MS2 scans (30,000 resolution, 2 × 10^5^ AGC, 45 ms maximal ion time, HCD, Stepped NCE (50, 100, 150), and 20 s dynamic exclusion).

Metabolomics mass spec data were converted into the mzXML format and processed using in-house JUMPm algorithm [30]. Briefly, metabolite peak features were detected for each sample and aligned among all the compared samples. Metabolites were annotated by matching the retention time, accurate mass/charge ratio, and MS/MS fragmentation data to our in-house authentic compound library or matching to downloaded experimental MS/MS library (MoNA, https://mona.fiehnlab.ucdavis.edu/) by accurate mass/charge ratio and MS/MS spectrum. The dot product algorithm was employed to score the identifications. Peak intensities were used for metabolite quantification. The data were further normalized for batch effect removal (LIMMA R package [29], followed by quantile normalization [31]. Quantification and statistical analysis were done by calculating fold changes and *p-*values between different groups using the LIMMA R package. MetaboAnalyst6.0 (www.metaboanalyst.ca) [32–34] was used to generate metabolite set enrichment analysis (MSEA) plots for pathway analysis.

### In vitro stable isotope tracing analysis by LC-MS/MS

One million cells were seeded and allowed to adhere overnight for each experiment. For the isotope tracing experiment, cells were washed with PBS and subsequently incubated with a medium containing ^13^C isotope-labeled tracer [either of U-^13^C glucose (Cambridge Isotope Limited: CLM-1396; 99%) or U-^13^C acetate (Cambridge Isotope Limited: CLM-440; 99%)] of interest supplemented with 5% dialyzed FBS (Gibco:A33820-01). For the U-^13^C glucose tracing condition, the medium contained 10 mM U-^13^C glucose [35] and unlabeled ^12^C- glutamine at 4 mM [35]. For the U-^13^C acetate [36, 37] tracing condition, the medium contained unlabeled ^12^C glucose at 10 mM and unlabeled ^12^C glutamine at 4 mM concentration. Cells were incubated under these conditions for 24 h. After incubation, cells were washed twice with ice-cold 1X PBS to remove residual medium. Metabolite extraction and LC-MS/MS methods were the same as untargeted metabolome profiling analyses except positive ion mode analysis for metabolomics was not pursued based on the metabolite targets of interested. Lipids were extracted in a similar way by freezing 100% isopropanol (LC-MS grade, Sigma-Aldrich) from one to three million cells and dried.

MS raw data was converted into the mzXML format and processed using in-house JUMPm algorithm. Briefly, the feature peaks of the isotopologues of all target metabolites were extracted and aligned among all the compared samples followed by natural isotope abundance correction and tracer impurity correction. The identified metabolites were validated by comparing with authentic standard compounds at the following parameters: mass/charge ratio, LC retention time, and MS/MS spectra. The peak intensities were used for quantification and calculating the labeling percentage of the isotopologues for each target metabolite.

### High-throughput drug combination screening and BRAID analysis

ES-8, SK ES-1, and EW-8 WT and *SLFN11*^−/−^ cells were seeded in 384-well plates with an automatic dispenser (WellMate, Thermo Scientific) at a density of 3000 cells/well. Following 24 h of incubation, concentrated compounds were pin transferred in the 30uL cell suspension using the Biomek FX Laboratory Automation Workstation (Beckman Coulter, Inc) resulting in a 1:1000 dilution. DMSO was used as negative control. A BRAID-format drug combination screen was performed using SN-38 as the anchor drug. SN-38 was assessed across seven concentrations in combination with ten concentrations of the partner compounds: FSG67 and SN-38 (self-control). Source plate stock concentrations were prepared at 1000× for a final assay dilution of: SN-38 (1 μM-0.05 nM) and FSG67 (200 μM–10 nM). Cells were treated in triplicate technical replicates across two biological replicates. After 72 h, cell viability was measured using CellTiter-Glo (Promega: G7573), and luminescence was recorded using an EnVision plate reader (PerkinElmer). Dose-response surfaces were modeled using the BRAID (Bivariate Response to Additive Interacting Doses) approach to quantify drug interactions [38, 39]. The κ (kappa) parameter indicates interaction type, where *κ* > 0 denotes synergy, *κ* = 0 indicates additivity, and *κ* < 0 indicates antagonism. IAE_50_ was calculated to assess the overall efficacy of each combination at 50% inhibition thresholds. IDMA and IDMB correspond to the EC_50_ values of the partner drug and the anchor drug, respectively. The statistical significance of interaction terms was evaluated based on 95% confidence intervals that were estimated using bootstrapping.

### Xenograft studies in mice

Athymic nude immunodeficient 8-week-old female mice were obtained from Charles River Laboratories (strain code: 490). All animal experiments were conducted in accordance with protocols approved by the Institutional Animal Care and Use Committee (IACUC), and efforts were made to minimize animal suffering. Mice were housed under a 12 h light/dark cycle and provided food and water ad libitum. To generate EWS orthotopic xenografts, ES-8 WT and ES-8 *SLFN11*^−/−^ cells were suspended in Matrigel (Corning, Catalogue no: 356234) at a concentration of 20,000 cells/μL. The cell suspensions were injected into the bone marrow as previously described in Stewart et al. [4], using a Hamilton syringe fitted with a 25-gauge needle. Mice were monitored daily, and experiments were discontinued upon ≥20% weight loss or evidence of poor health. Once tumors became palpable and reached a diameter of ~20 mm, mice were euthanized and the tumors grown surrounding the femur were harvested and used for downstream analyses.

### Preparation for tumor extracts

Metabolite extraction from tumor extracts was done following the method described by Patel et al. [40]. Briefly, frozen tissue (150–200 mg) from tumor grown surrounding the femur was ground with 0.1 M HCl/methanol (2:1 vol wt) at 40 °C followed by extraction with ice-cold ethanol. The supernatant was clarified by centrifugation, lyophilized, and resuspended in 500 µL of phosphate-buffered (25 mM, pH-7) D_2_O solution containing 3-(trimethylsilyl) propionic-2,2,3,3-d_4_ acid (0.05 wt%) (Sigma).

### NMR spectroscopy of tumor extracts for ^1^H NMR

¹H NMR spectra of tumor extracts were acquired at 298 K on a 600 MHz Bruker Avance NEO NMR spectrometer (Bruker BioSpin, Billerica, MA) equipped with a 5 mm TCI cryoprobe. Acquisition parameters included a spectral width of 13.0 ppm, 16k data points, a relaxation delay of 2 s, an acquisition time of 1.1 s, and 64 scans. The residual H₂O signal was suppressed using the excitation sculpting technique [41]. Free induction decays (FIDs) were Fourier-transformed and analyzed using Bruker Topspin 4.3.0 software. The peak intensity of different metabolites was measured, and choline-containing compounds were identified based on their characteristic chemical shifts: phosphocholine (PCh, 3.226 ppm), glycerophosphocholine (GPC, 3.235 ppm), and free choline (Cho, 3.208 ppm) [42].

### Statistical analysis

MetaboAnalyst 6.0, R software and MATLAB (R2023b) were used to conduct statistical analysis of metabolomics data. For additional data analysis, GraphPad Prism V10.4.0 was used. Data visualization used GraphPad Prism, MATLAB, and R software. Descriptions of individual statistical analyses can be found in the figure legends. *p* < 0.05 was considered statistically significant.

## Results

### *SLFN11* expression is upregulated in EWS

**Fig. 1:**
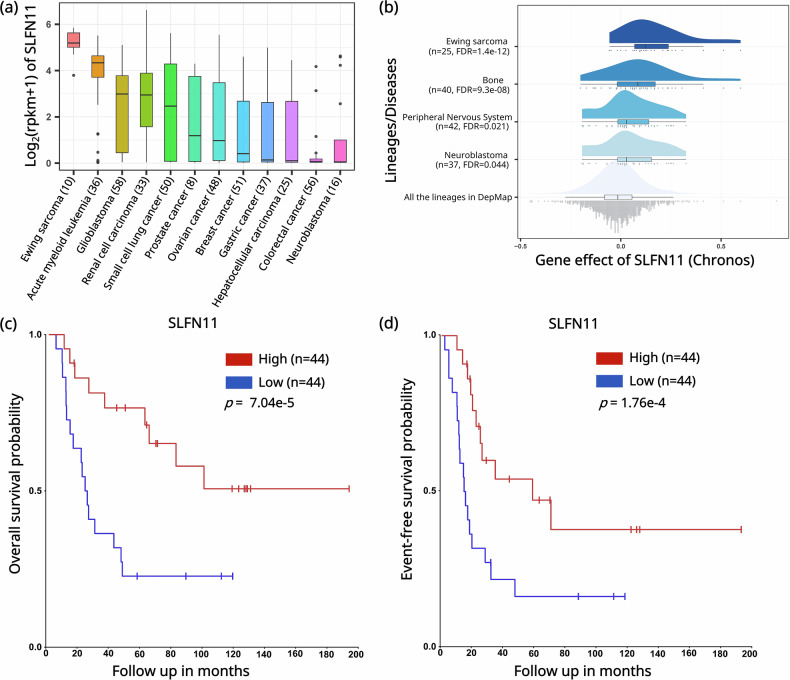
*SLFN11* is highly expressed in EWS and is associated with improved prognosis. **a** *SLFN11* expression across 428 primary tumors. Box-and-whisker plots show the distribution of mRNA expression for each subtype, ordered by the median *SLFN11* expression level (line), the inter-quartile range (box) and up to 1.5x the inter-quartile range (bars). Sample numbers (n) are indicated in parentheses. **b** Gene dependency analysis of *SLFN11* across tissue lineages and primary disease types of all cell lines using Chronos dependency scores from the DepMap project. Kaplan-Meier survival analysis from the ESCLA database comparing patients with high (red line) versus low (blue line) *SLFN11* expression. Expression levels were dichotomized based on the median mRNA expression values. **c** Overall survival analysis of patients with high (*n* = 44) and low (*n* = 44) *SLFN11* expression using a median cutoff of 464.5 (*p*-value = 7.04e-5). **d** Event-free survival analysis of patients with high (*n* = 44) and low (*n* = 44) *SLFN11* expression using a median cutoff of 464.5 (*p*-value = 1.76e-4). *n* is number of patients.

CCLE is a compilation of gene expression, chromosomal copy number and next-generation sequencing data from 947 human cancer cell lines [10, 23]. Transcriptomic analysis from the CCLE reveals that *SLFN11* is broadly expressed across multiple cancer types, with EWS showing consistently elevated expression levels (Fig. 1a). The Cancer Dependency Map (DepMap) is a functional genomics resource that integrates CRISPR and RNAi screening data with molecular profiles across diverse cancer cell lines to identify essential genes and lineage-specific dependencies [43]. Analysis of EWS data derived from DepMap functional genomics further revealed that *SLFN11* had a Chronos gene effect score close to zero (*p*FDR = 1.4e-12) in EWS compared to all cell lineages (Fig. 1b). In DepMap, Chronos scores near zero are consistent with non-essential genes and indicate that *SLFN11* knockout does not compromise cell viability of EWS cell lines [44]. Previous analyses of EWS patient cohorts and comparative datasets from The Cancer Genome Atlas Program (TCGA) have demonstrated that higher *SLFN11* expression is associated with improved therapeutic sensitivity and patient survival [11]. Our analysis of the EWS cohort from the Ewing Sarcoma Cell Line Atlas (ESCLA) [24] database also revealed that elevated *SLFN11* expression is associated with improved overall (*p* = 7.04e-5) and event-free (*p* = 1.76e-4) patient survival (Fig. 1c, d). The ESCLA is a multi-omics resource of 18 EWS cell lines with inducible *EWSR1-ETS* knockdown, enabling analysis of fusion-driven gene regulation and molecular heterogeneity [24]. Together, these analyses reveal that *SLFN11* is highly expressed in EWS and higher expression levels are associated with improved therapeutic response and patient survival, while its loss does not impact cell viability.

### *SLFN11* knockout is associated with downregulation of the mitochondrial *GPD2*

**Fig. 2:**
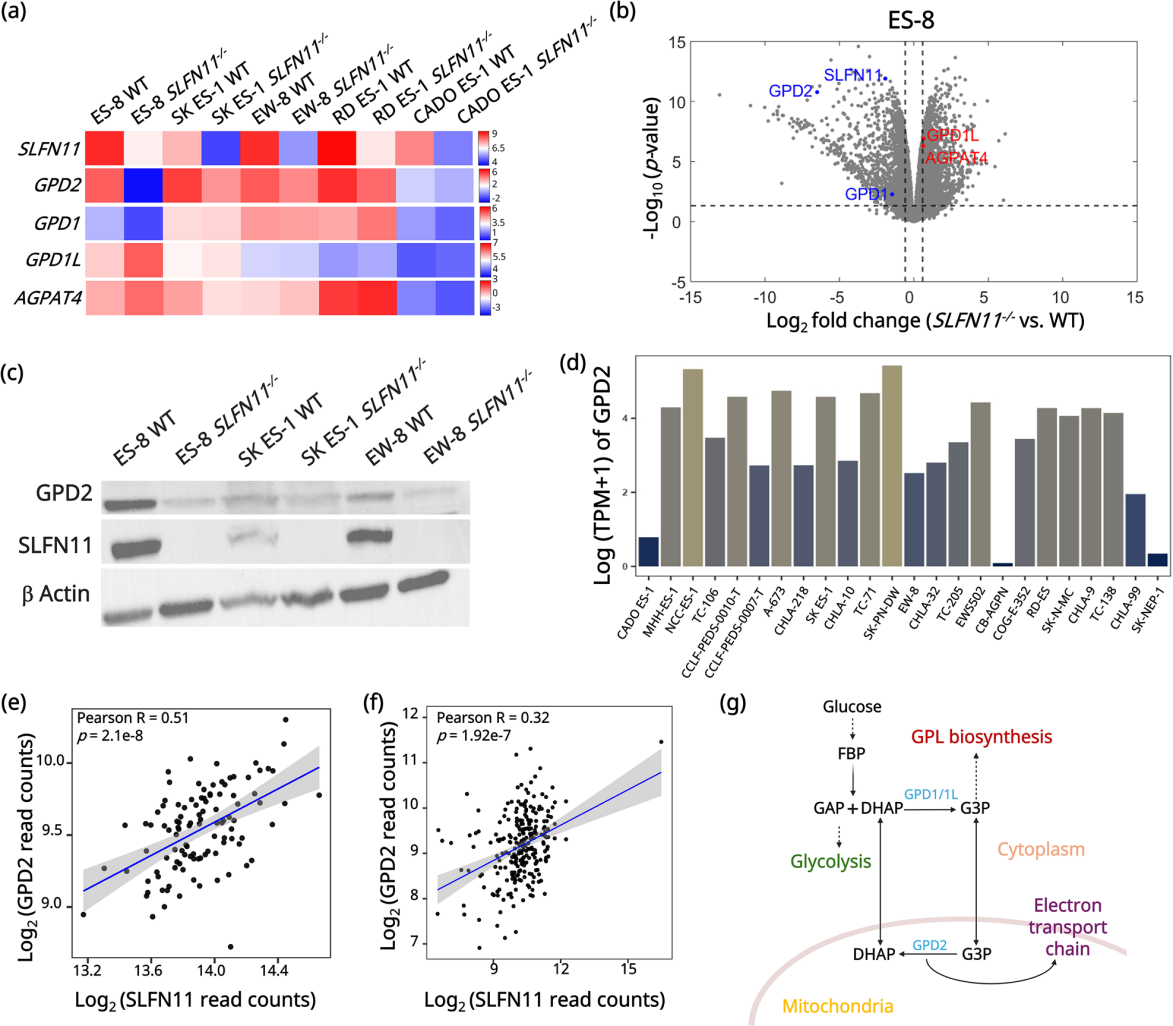
Knockout of *SLFN11* reprograms the G3PS through downregulation of mitochondrial *GPD2.* **a** Heatmap showing relative *mRNA expression* levels from RNA-seq analysis for *SLFN11*, *GPD2*, *GPD1*, *GPD1L*, and *AGPAT4* across WT and *SLFN11*^*−/−*^ EWS cell lines ES-8, SK ES-1, EW-8, RD ES-1, and CADO ES-1. Color scale represents relative gene expression. Each gene is independently scaled, and the accompanying color bar represents the relative expression range for that gene across EWS cell lines. **b** Volcano plot showing differentially expressed genes in ES-8 *SLFN11*^−/−^ versus ES-8 WT cell line from RNA-seq analysis. Red dots indicate significantly upregulated genes involved in G3PS (glycerol-3-phosphate shuttle), while blue dots represent significantly downregulated genes. **c** Western blot analysis of GPD2 (mitochondrial glycerol-3-phosphate dehydrogenase) and SLFN11 protein expression in ES-8 WT and *SLFN11*^−/−^, SK ES-1 WT and *SLFN11*^−/−^, and EW-8 WT and *SLFN11*^−/−^ cell lines. β-Actin serves as a loading control. **d** Bar plot showing GPD2 mRNA expression (log₂ [TPM + 1]) across 24 EWS cell lines from the DepMap dataset. Full-length uncropped Western blots corresponding to this figure are provided in the Supplemental Material. Correlation analysis between log₂-transformed *SLFN11* and *GPD2* read counts across EWS tumors in the **e** ESCLA (Ewing Sarcoma Cell Line Atlas) dataset (R = 0.51, *p* = 2.1e-08) and **f** DepMap dataset (R = 0.32, *p* = 1.92e-07). R represents the Pearson correlation coefficient. Statistical significance was defined as p < 0.05. **g** Schematic of the G3PS showing cytosolic conversion of DHAP (dihydroxyacetone phosphate) to G3P (glycerol-3-phosphate) via *GPD1/1L* (cytosolic glycerol-3-phosphate dehydrogenase), followed by mitochondrial oxidation of G3P by *GPD2*, transferring electrons to the ETC (electron transport chain) to support OXPHOS (oxidative phosphorylation). G3P also serves as the backbone for GPL (glycerophospholipid) biosynthesis in the cytosol.

To investigate *SLFN11*^−/−^ associated transcriptional changes more broadly, we performed RNA-sequencing (RNA-seq) analysis across patient derived EWS cell lines (Table S1). To capture disease-relevant diversity, we selected cell lines harboring the hallmark *EWSR1- ETS* fusions, namely *EWSR1-FLI1* type I (EW-8) and type II (ES-8, SK ES-1, RD ES-1), and *EWSR1-ERG* (CADO ES-1), which are the principal oncogenic drivers of EWS. Principal component analysis (PCA) revealed clear segregation between WT and *SLFN11*^−/−^ cell lines along first principal component (PC1) (Fig. S1a-e). RNAseq analysis further revealed a consistent downregulation of *GPD2* in the following EWS *SLFN11*^−/−^ cell lines: ES-8 (Log_2_FC = −6.5, *p*FDR = 5.45e-9) (Fig. 2a,b), SK ES-1 (Log_2_FC = −1.33, *p*FDR = 4.5e-6) (Figs. 2a, S1f), EW-8 (Log_2_FC = −0.73, *p*FDR = 5.27e − 2) (Fig. 2a, S1g), and RD ES-1 (Log_2_FC = −0.89, *p*FDR = 2.17e-7) (Fig. 2a, S1h). However, *GPD2* expression was unaffected in CADO ES-1 cell line between WT and *SLFN11*^−/−^ cell lines (Fig. 2a, S1i). The cytosolic isoforms of glycerol-3-phosphate dehydrogenase, *GPD1L* and *GPD1*, were also differentially upregulated across the *SLFN11*^*−/−*^ EWS cell lines; *GPD1L* expression was significantly elevated in ES-8 *SLFN11*^*−/−*^ cell line (Log_2_FC = 0.65, *p*FDR = 2.14e-6) (Fig. 2a,b), while *GPD1* expression was elevated in RD ES-1 *SLFN11*^*−/−*^ cell line (Log_2_FC = 0.66, *p*FDR = 4.2e-2); (Fig. 2a, S1h).

To confirm the transcriptomics findings, we performed Western blot analysis of *GPD2* expression in selected EWS cell lines (Fig. 2c, S1j). A decrease in GPD2 protein was evident in *SLFN11*^*−/−*^ ES-8 and EW-8, while SK ES-1 exhibited a non-significant downward trend in GPD2 expression (Fig. 2c, S1j). Analysis of *GPD2* expression across multiple cancer subtypes using CCLE patient-derived datasets revealed no significant variation among tumor types (Fig. S1k).

Next, we examined publicly available datasets to assess broader GPD2 expression correlations. Quantitative analysis of GPD2 mRNA expression across 24 EWS cell lines from the DepMap database revealed heterogeneous expression (Fig. 2d), indicating substantial variability in GPD2 abundance across EWS models. However, analysis of the ESCLA revealed a strong positive correlation between *SLFN11* and *GPD2* expression (Pearson R = 0.51, *p* = 2.1e-8) (Fig. 2e). Similarly, across EWS cell lines in the DepMap database, *SLFN11* and *GPD2* expressions were positively correlated (Pearson R = 0.32, *p* = 1.92e-7) (Fig. 2f).

These findings support a potential regulatory or functional relationship between *SLFN11* and *GPD2. GPD2* plays a central role in the glycerol-3-phosphate shuttle (G3PS), a key shuttle system connecting glycolysis, lipid metabolism, and redox homeostasis (Fig. 2g) [22, 45]. GPD2 catalyzes the oxidation of G3P to dihydroxyacetone phosphate (DHAP), a glycolytic intermediate in mitochondria. This interconversion transfers electrons to the electron transport chain (ETC). In the cytosol, *GPD1/GPD1L* reduces DHAP to G3P (Fig. 2g) [22, 45, 46]. The G3PS enables redox coupling between cytosol and mitochondria by exchanging DHAP and G3P.

### *GPD2* downregulation elevates G3P levels in *SLFN11*-knockout cells

Building on these findings, we next investigated the metabolic consequences of *SLFN11* loss and the associated downregulation of *GPD2* by performing metabolomic profiling of *SLFN11*^*−/−*^ and WT EWS cell lines. To achieve this, we used LC-MS to profile a wide range of intracellular metabolites without predefined targets, enabling the identification of global metabolic alterations and pathway-level changes associated with *SLFN11* knock out. To maintain consistency and comparability across all samples, we performed batch effect removal and applied quantile normalization (Fig. S2a-d). PCA was utilized to reduce data dimensionality and visualize patterns within the metabolomic profiles (Fig. S2e, f). The PCA results revealed a clear separation between the *SLFN11*^*−/−*^ and the WT cell lines, indicating distinct metabolic states induced by *SLFN11* knockout in ES-8 (Fig. S2e) and SK ES-1 (Fig. S2f) cell lines.

**Fig. 3:**
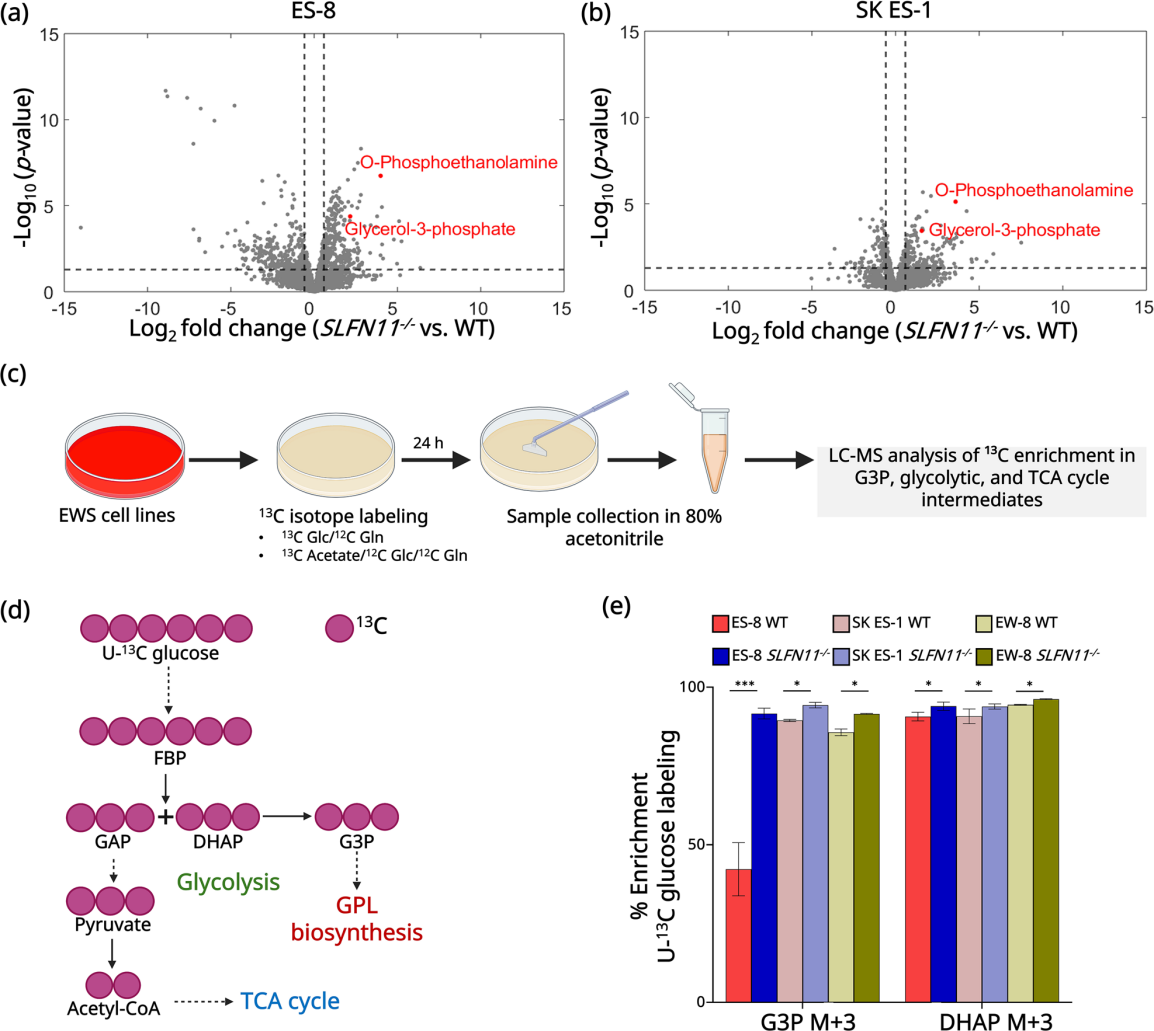
*SLFN11* knockout promotes G3P accumulation in EWS cells. Volcano plots illustrating differentially abundant metabolites between *SLFN11*^*−/−*^ and WT cells in (**a**) ES-8 and (**b**) SK ES-1 cell lines. Red dots indicate significantly upregulated metabolites involved in GPL biosynthesis. **c** Schematic overview of U-¹³C glucose tracing using LC-MS to assess ¹³C enrichment in downstream metabolic intermediates across cell lines. Glc and Gln correspond to glucose and glutamine, respectively. **d** Schematic showing U-¹³C glucose-derived carbon incorporation into glycolysis and GPL biosynthesis. Purple circles represent ¹³C-labeled carbon atoms. **e** Bar graph showing percentage differences in ¹³C enrichment of the M + 3 isotopologue for DHAP and G3P in WT and *SLFN11*^*−/−*^ ES-8, SK ES-1 and EW-8 cell lines following 24 h labeling with U-¹³C glucose. G3P, glycerol-3-phosphate; DHAP, dihydroxyacetone phosphate. Data represent mean ± SD; *n* = 5. Statistical analysis was performed using a paired two-tailed Student’s *t* test for each comparison. *** *p* < 0.001, * *p* < 0.05. SD standard deviation; *n* number of replicates.

To investigate the metabolic changes associated with *SLFN11* knockout, we generated volcano plots based on unsupervised clustering analysis (Fig. 3a, b, Table S2, S3). These plots highlighted considerable metabolic alterations in *SLFN11*^*−/−*^ cell lines compared to WT, with ES-8 showing 1,811 downregulated and 1,512 upregulated metabolites, and SK ES-1 showing 1,211 downregulated and 1,611 upregulated metabolites (Fig. 3a, b, Table S2-S3). Notably, G3P showed a marked increase in both ES-8 (Log_2_FC = 2.28, *p*FDR = 1.43e-6) (Fig. 3a, Table S2) and SK ES-1 (Log_2_FC = 1.5, *p*FDR = 7.33e-4) (Fig. 3b, Table S3) *SLFN11*^−/−^ cell lines compared to their respective WT cell lines.

To further explore this observation, we performed in vitro stable isotope tracing using U-¹³C glucose (Fig. 3c). Glucose was supplied at a concentration of 10 mM, a standard concentration in isotope tracing studies that ensures effective label incorporation [35]. This approach enables the quantification of glycolytic flux into G3P, linking glucose metabolism to GPL synthesis as illustrated in Fig. 3d. Increased ¹³C enrichment in G3P reflects enhanced channeling of glucose-derived carbon toward lipid biosynthesis. Uniformly labeled ¹³C-glucose generates predominantly M + 3 isotopologues of G3P, signifying complete incorporation of three ¹³C atoms into its carbon backbone [47]. Using this approach, we observed that ES-8 *SLFN11*^−/−^ exhibited a 2.17-fold higher ^13^C labeling in G3P M + 3 isotopologue compared to ES-8 WT cell line (*p* = 5.7e-4), (Fig. 3e, Table S4) confirming elevated G3P enrichment. Similarly, SK ES-1 and EW-8 cell lines exhibited modest but significant increase in G3P labeling (*p* = 1.52e-4 and 6.96e-4, respectively) in the *SLFN11*^−/−^ cell lines compared to the respective WT cell lines (Fig. 3e, Table S5, S6). These labeling patterns align with our Western blot analysis (Fig. 2c, S1j), which shows a pronounced decrease in *GPD2* in ES-8 and modest downregulation in SK ES-1 and EW-8 following *SLFN11* knockout. DHAP was upregulated in ES-8 *SLFN11*^−/−^ cell line by 1.04-fold (*p* = 4e-2), in SK ES-1 *SLFN11*^*−/−*^ cell line by 1.03-fold (*p* = 1.63e-2), and in EW-8 *SLFN11*^*−/−*^ cell line by 1.07-fold (*p* = 1.2e-4) compared to their respective WT cell lines (Fig. 3e, Table S4-S6).

We also observed an upregulation of O-phosphoethanolamine in ES-8 *SLFN11*^*−/−*^ (Log_2_FC = 3.9, *p*FDR = 9.07e-10) (Fig. 3a, Table S2) and SK ES-1 *SLFN11*^*−/−*^ (Log_2_FC = 3.63, *p*FDR = 1.15e-8) (Fig. 3b, Table S3) cell lines compared to corresponding WT cell lines. O-Phosphoethanolamine is an essential metabolite in phosphatidylethanolamine (PE) biosynthesis [48]. O-phosphoethanolamine is a key intermediate in the CDP ethanolamine pathway, which leads to the synthesis of phosphatidylethanolamine, one of the most abundant phospholipids in cellular membranes. In this pathway, ethanolamine is first phosphorylated to form O-phosphoethanolamine, which is then converted to CDP ethanolamine and ultimately incorporated into PE [48]. Elevated O-phosphoethanolamine levels indicate an increased demand for membrane biogenesis, which has been associated with tumor aggressiveness and altered signaling in cancer cells [49, 50].

Collectively, these findings revealed that GPD2 downregulation in *SLFN11*^*−/−*^ cells leads to G3P accumulation and altered metabolic flux involving the G3PS. A recently published study has reported that GPD2 knockdown results in G3P accumulation and facilitates the synthesis of complex lipids in kidney cancer [22]. Our findings showing elevated G3P levels suggest a potential shift toward lipid biosynthesis in *SLFN11*^*−/−*^ EWS cells, as G3P serves as the backbone for the synthesis of key GPL species, including PE, phosphatidylcholine (PC), phosphatidylinositol (PI), phosphatidylserine, phosphatidylglycerol (PG), cardiolipin, phosphatidic acid (PA), diacylglycerol (DAG), and triacylglycerol via sequential acylation with fatty acids [22, 45, 51].

### *SLFN11* knockout promotes GPL biosynthesis via *GPD2* downregulation

**Fig. 4:**
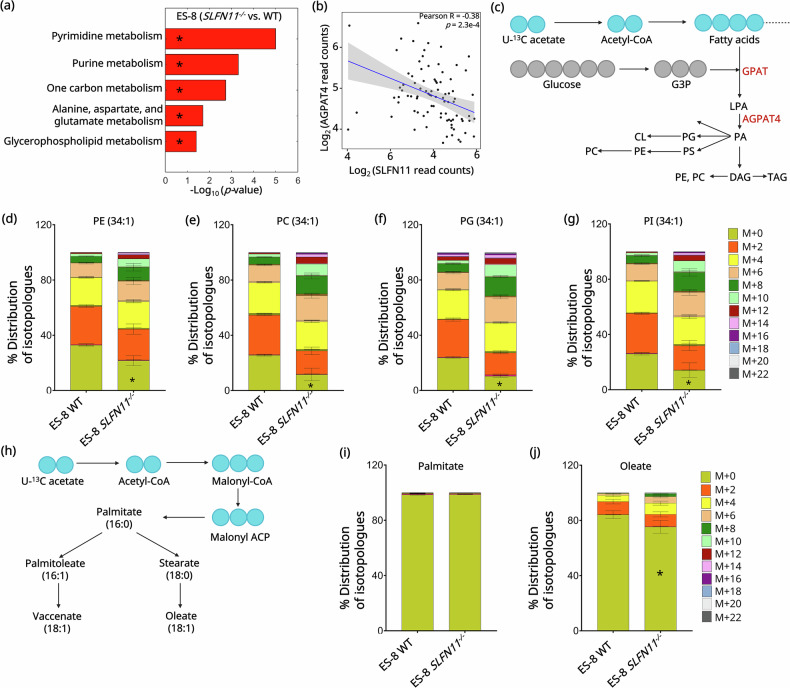
*SLFN11* knockout increases GPL biosynthesis in EWS. **a** MSEA-based pathway enrichment analysis identifying upregulated metabolic pathways in ES-8 *SLFN11*^*−/−*^ cell line. Pathways with –log₁₀(FDR) ≥ 0.5 were considered enriched and ranked by significance. Asterisks mark statistically significant pathways. **b** Correlation analysis of log₂-transformed *SLFN11* and *AGPAT4* expression across EWS tumors using patient-derived microarray data [25] from the Gene Expression Omnibus (GEO) database. (*R* = −0.38, *p* = 2.3e-04). R represents the Pearson correlation coefficient. Statistical significance was defined as *p* < 0.05. **c** Schematic illustrating showing U-¹³C acetate incorporation into the GPL biosynthesis pathway. Cyan circles represent ¹³C-labeled acetate-derived carbons incorporated into fatty acid chains. Gray circles denote the synthesis of G3P backbone. Enzymes involved in GPL biosynthesis are highlighted in maroon. Isotopologue distribution of U-¹³C acetate-labeled GPL species, PE (34:1) **d**, PC (34:1) **e**, PG (34:1) **f**, and PI (34:1) **g** in ES-8 WT and *SLFN11*^*−/−*^ cells. Bars represent the relative abundance of individual mass isotopologues (M + 2 to M + 22), corresponding to successive incorporation of ¹³C-labeled acetate units during GPL synthesis. A color scale denoting isotopologue species (M + 0 to M + 22) is included alongside the figure. Data represent mean ± SD (*n* = 5). Statistical comparisons were performed using paired two-tailed Student’s *t* test for each comparison and the corresponding *p*-values are reported in Table S9. The asterisk denotes statistical significance for the M₀ isotopologue (*p* < 0.05). SD, standard deviation; *n*, number of replicates. **h** Schematic of U-¹³C acetate tracing into de novo fatty acid synthesis. Cyan circles represent ¹³C-labeled acetate-derived carbons incorporated during elongation. The diagram illustrates labeling flow into saturated and monounsaturated fatty acid species. Distribution of U-¹³C acetate-labeled isotopologues in palmitate (**i**) and oleate (**j**) in ES-8 WT and *SLFN11*^*−/−*^ cells. Color code for isotopologues (M + 0 to M + 22) is shown adjacent to panel. Bars represent mean ± SD (*n* = 5). Statistical comparisons were performed using paired two-tailed Student’s *t* test for each comparison. SD standard deviation; *n* number of replicates.

Given the association between *SLFN11* knock out, GPD2 downregulation, and G3P accumulation, we next investigated the metabolic pathways altered by loss of *SLFN11*. To achieve this, we performed metabolite set enrichment analysis (MSEA) using MetaboAnalyst 6.0 [32, 34]. The analysis identified GPL biosynthesis as a key pathway upregulated with *SLFN11* knockout in both ES-8 (Fig. 4a, Table S7) and SK ES-1 (Fig. S2g, Table S8) cell lines. While the findings in SK ES-1 achieved statistical significance (*p* = 9.30e-3), the results in ES-8 exhibited a trend (*p* = 5.71e-2).

Transcriptomics analysis demonstrated significant upregulation of *AGPAT4* (Log_2_FC = 0.63, *p*FDR = 6.37e-6), a critical gene involved in GPL biosynthesis, [22, 52, 53] in the ES-8 *SLFN11*^−/−^ cell line (Fig. 2a, b). To validate this observation in patient-derived data, we analyzed EWS tumor microarray profiles from the Gene Expression Omnibus (GEO) database, which revealed a significant inverse correlation between *SLFN11* and *AGPAT4* expression (Pearson *R* = −0.38, *p* = 2.3e-4) (Fig. 4b).

To further investigate the observed upregulation of GPL biosynthesis, we performed stable isotope tracing using U-¹³C acetate. Acetate was supplied at 0.4 mM, a physiologically relevant concentration frequently used in isotope tracing studies to capture acetate utilization in metabolic pathways [36, 37]. Acetate serves as a key carbon source for fatty acid biosynthesis by providing two-carbon units through cytosolic acetyl-CoA, and stable isotope tracing with U-¹³C-acetate enables quantification of its incorporation into GPLs [36]. Each acetate unit provides two ¹³C atoms, producing M + 2, M + 4, M + 6, and higher isotopologues through stepwise incorporation during fatty acid elongation as illustrated in Fig. 4c. Elevated levels of these isotopologues indicate enhanced de novo lipid biosynthesis from acetate [36]. Given the significantly higher ¹³C-glucose-derived G3P labeling and consistent metabolic profile observed in patient-derived ES-8 cells (Fig. 3e), we selected this cell line as an optimal system for further U-¹³C acetate tracing analysis to assess GPL synthesis. LC-MS analysis revealed significant incorporation of ¹³C- acetate-derived carbons into GPL species in ES-8 *SLFN11*^*−/−*^ compared to ES-8 WT cell line (Fig. 4d-g, Table S9). The total ¹³C labeling of PE was higher in *SLFN11*^*−/−*^ (78%) compared to WT ES-8 cell line (67%) (*p* = 2.72e-03) (Fig. 4d, Table S9). Similarly, in PC, 88.4% labeling was observed in *SLFN11*^*−/−*^ cell line, compared to 74.4% in WT (*p* = 3.18e-3) (Fig. 4e, Table S9). In PG ~ 90% of the lipid pool was labeled in *SLFN11*^*−/−*^ cell line versus 76% in WT (*p* = 2.21e-9) (Fig. 4f, Table S9). PI showed 86% labeling in *SLFN11*^*−/−*^ versus 74% in WT (*p* = 1.03e-2) (Fig. 4g, Table S9). These findings suggest that *SLFN11* knockout enhances ^13^C-acetate flux into membrane lipid biosynthesis, indicating a shift toward increased reliance on acetate for de novo GPL biosynthesis. The distributions of all isotopologues of these lipid species are presented in Fig. 4d-g and Table S9.

Additionally, we assessed fatty acid labeling using U-¹³C acetate, as illustrated in Fig. 4h. This tracer enables evaluation of fatty acid elongation by tracking the incorporation of ¹³C-labeled acetate units. *SLFN11*^*−/−*^ cells exhibited increased labeling in monounsaturated fatty acids (MUFAs). No significant difference of ¹³C labeling in palmitate (saturated fatty acid) was observed between ES-8 *SLFN11*^*−/−*^ and ES-8 WT cell lines (Fig. 4i), whereas increased labeling of oleate (unsaturated fatty acid) was seen in ES-8 *SLFN11*^*−/−*^ (~25%) compared to ES-8 WT cell line (~ 15%) (Fig. 4j) (*p* = 1.19e-2). Increased synthesis of monounsaturated fatty acids enhances membrane fluidity, which has been linked to greater tumor aggressiveness and adaptability to metabolic stress [54–56]. Taken together, these results highlight the metabolic reprogramming driven by *SLFN11* knockout, with a marked shift toward unsaturated fatty acid biosynthesis pathways.

### *SLFN11* knockout sensitizes EWS cells to combined DNA damage and lipid biosynthesis inhibition

**Fig. 5:**
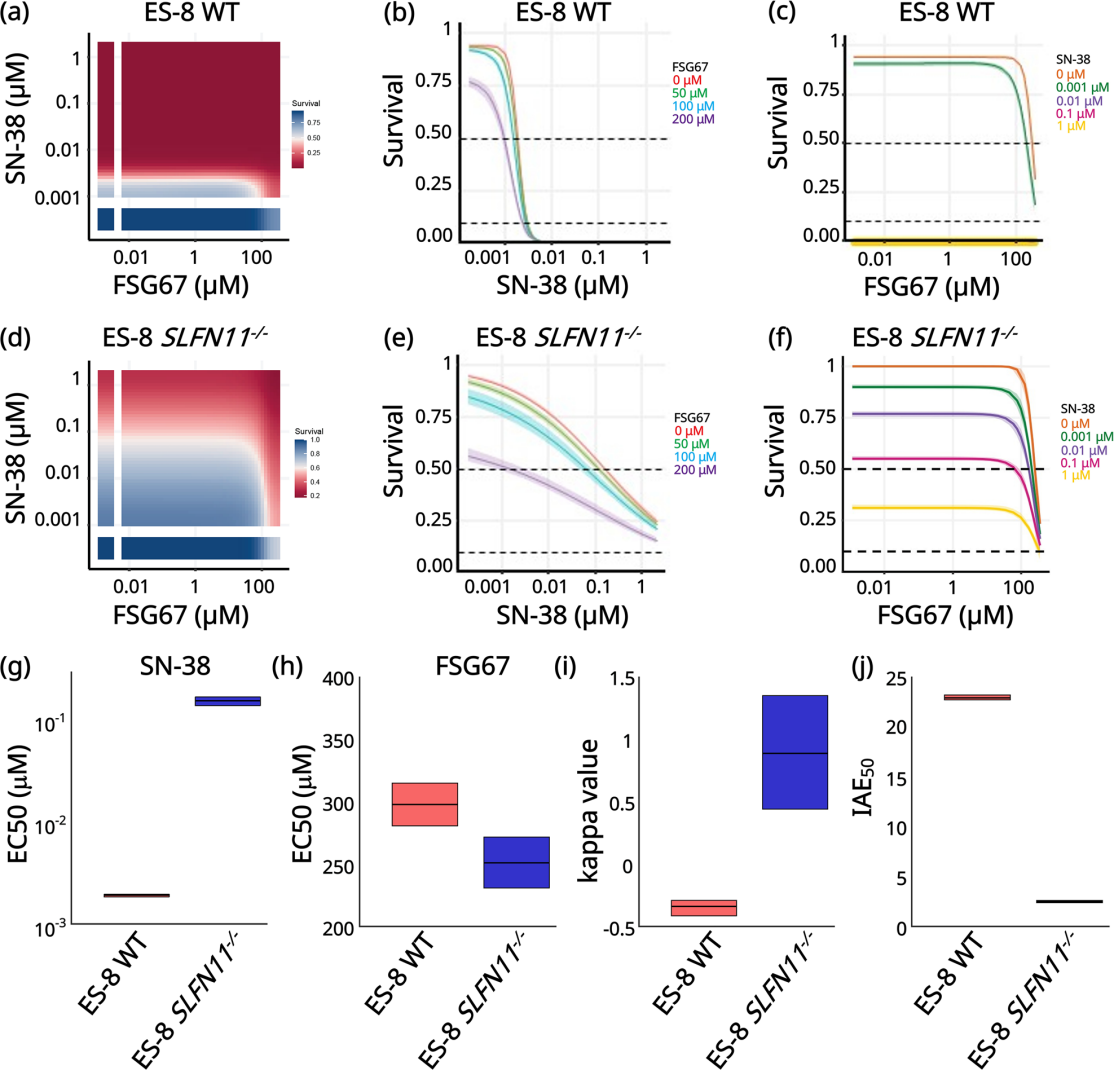
*SLFN11* knockout sensitizes EWS cells to combined DNA damage and *GPAT1* inhibition. BRAID analysis from two pooled bioreplicate experiments testing ES-8 WT and *SLFN11*^*−/−*^ cells with SN-38 and FSG67 for 72 h. **a** Response surface plot for ES-8 WT cells. Dose response curve for SN-38 (**b**) and FSG67 (**c**) in ES-8 WT cells. **d** Response surface plot for ES-8 *SLFN11*^*−/−*^ cells. Dose response curve for SN-38 (**d**) and FSG67 (**e**) in ES-8 *SLFN11*^*−/−*^ cells. Quantitative BRAID analysis of the interaction between SN-38 and FSG67 in ES-8 WT and *SLFN11*^*−/−*^ cells. Integrated Drug Mean Activity, reported as IDMB for SN-38 (**g**) and IDMA for FSG67 (**h**), representing the BRAID-derived EC_50_ values for the two single agents. **i** *κ* (kappa) values, where values = 0 indicate additivity; < 0 indicate antagonism; 0 indicate synergy. Results were obtained from pooling two independent replicates and are plotted with 95% confidence intervals. **j** Index of Achievable Efficacies (IAE), shown as IAE_50_, quantifying the magnitude of combination benefit at 50% inhibition in ES-8 WT and *SLFN11*^*−/−*^ cells.

To explore whether the elevated GPL pathway in *SLFN11*^*−/−*^ cells could be therapeutically exploited, we treated ES-8, SK ES-1 and EW-8 WT and *SLFN11*^*−/−*^ cells with SN-38, a potent topoisomerase I inhibitor [57] with *SLFN11*-dependent activity, [15, 17] combined with FSG67, a selective glycerol-3-phosphate acyltransferase 1 (GPAT1) inhibitor [22]. GPAT1 is a key enzyme in de novo GPL biosynthesis that supports membrane lipid production [22]. Cell viability was assessed after 72 h of drug treatment using CellTiter-Glo, and the results were analyzed using the BRAID response surface model [38, 39] (Fig. 5, S3, Table S10).

In ES-8 WT cells, SN-38 was highly potent as a single agent (EC_50_ = 1.92 nM; 95% CI [1.85-1.97 nM]) (Fig. 5b,g, Table S10), consistent with previous reports of *SLFN11*-dependent cytotoxicity [15]. However, co-treatment with FSG67 in these cells (EC_50_ = 298 µM; 95% CI [281–315 µM]) (Fig. 5c,h, Table S10) resulted in limited additional benefit and was antagonistic (BRAID *κ* = −0.338; 95% CI [(-0.41) − (−0.29)]) (Fig. 5a,i Table S10). In contrast, in ES-8 *SLFN11*^*−/−*^ cells, SN-38 was nearly 100 times less potent (EC_50_ = 0.157 µM; 95% CI [0.140-0.171 µM]) (Fig. 5e,g, Table S10) but FSG67 was more potent (EC_50_ = 251 µM; 95% CI [231–272 µM]) (Fig. 5f, h), and the combination of SN-38 and FSG67 was synergistic (BRAID *κ* = 0.889, 95% CI [0.44–1.35]) (Fig. 5d,i Table S10). Dose-response plots from the prospective of SN-38 clearly show that in *SLFN11*^*−/−*^ cells, FSG67 alone exhibits higher efficacy (Fig. 5f) and reduces the SN-38 EC_50_ when used in combination (Fig. 5d, Table S10).

Similarly, SK ES-1 WT (EC_50_ = 0.59 nM; 95% CI [0.36-0.59 nM]) (Fig. S3e,m, Table S10) and EW-8 WT (EC_50_ = 3.54 nM; 95% CI [3.50–3.67 nM]) (Fig. S3g,m, Table S10) cells showed strong sensitivity to SN-38. Similar to the findings of ES-8 cell lines, co-treatment with the FSG67 did not enhance the cytotoxic effect of SN-38 in the WT models. In SK ES-1 WT cells, FSG67 alone showed an EC_50_ of 293 µM (95% CI [228- 446 µM]) (Figure S3i,n, Table S10); and in EW-8 WT cells, EC_50_ = 840 µM (95% CI [535–1411 µM]) (Figure S3k,n, Table S10). Combination treatment resulted in limited additional benefit and was quantitatively antagonistic in all WT lines, as indicated by negative BRAID κ values (SK ES-1 WT: *κ* = −0.146; 95% CI [(−0.44)−0.43] (Fig. S3a,o, Table S10); EW-8 WT: *κ* = −0.370; 95% CI [(−0.52) − (−0.25)]) (Fig. 5c,o; Table S10). In contrast, in SK ES-1 *SLFN11*^*−/−*^ cells, SN-38 was nearly 85 times less potent (EC_50_ = 0.085 µM; 95% CI [0.072- 0.094 µM]) (Fig. S3f,m) but FSG67 was more potent (EC_50_ = 364 µM; 95% CI [225-561 µM]) (Fig. S3j,n, Table S10), and the combination of SN-38 and FSG67 was synergistic, although the effect did not reach statistical significance (BRAID κ = 0.990; 95% CI [−0.33−2.25]) (Fig. S3b, o, Table S10). In case of EW-8 *SLFN11*^*−/−*^cell line, a similar trend was observed SN-38 sensitivity with an EC_50_ of 13.29 nM (95% CI [10.63−16.01 nM]) (Figure S3h,m, Table S10) and FSG67 sensitivity (EC_50_ of 300 µM (95% CI [218-941 µM]) (Figure S3l,n, Table S10). The combination of SN-38 and FSG67 was synergistic, however, the interaction did not achieve statistical significance (BRAID κ = 0.221; 95% CI [−0.53−3.62]) (Fig. S3d, o, Table S10). The overall combined efficacy was lower in *SLFN11*^*−/−*^ cells than that in WT cells, as reflected by a reduced Index of Achievable Efficacies, IAE_50_ (ES-8 *SLFN11*^*−/−*^: 2.51, 95% CI [2.40-2.62] (Fig. 5j; Table S10); SK ES-1 *SLFN11*^*−/−*^: 3.36, 95% CI [3.17-3.58] (Fig. S3p; Table S10); EW-8 *SLFN11*^*−/−*^: 8.37, 95% CI [7.63-9.36]) (Fig. S3p; Table S10), compared to WT (ES-8 WT: 22.92, 95% CI [22.72-23.21] (Fig. 5j; Table S10); SK ES-1 WT: 42.16, 95% CI [42.16-53.32] (Fig. S3p; Table S10); EW-8 WT: 16.61, 95% CI [16.35-16.67]) (Fig. S3p; Table S10), indicating that loss of *SLFN11* diminishes the potency of SN-38 even under combination treatment.

Collectively, these findings indicate that *SLFN11* knockout selectively sensitizes ES-8 and SK ES-1 cells to the combined inhibition of DNA damage repair and lipid biosynthesis, thereby uncovering a therapeutically exploitable metabolic vulnerability. Previous studies have reported that *SLFN11* expression in EW-8 cells is approximately 0.59-fold lower relative to ES-8, which may explain the weaker interaction observed with the drug combination in this cell line. Consistent with this, SN-38 showed a more pronounced shift, and the increase in IAE_50_ are greater in ES-8 and SK ES-1 *SLFN11*^*−/−*^ cells, suggesting that the *SLFN11*-dependent phenotype is stronger in these models.

### *SLFN11* loss alters choline metabolism in EWS tumor xenografts

**Fig. 6:**
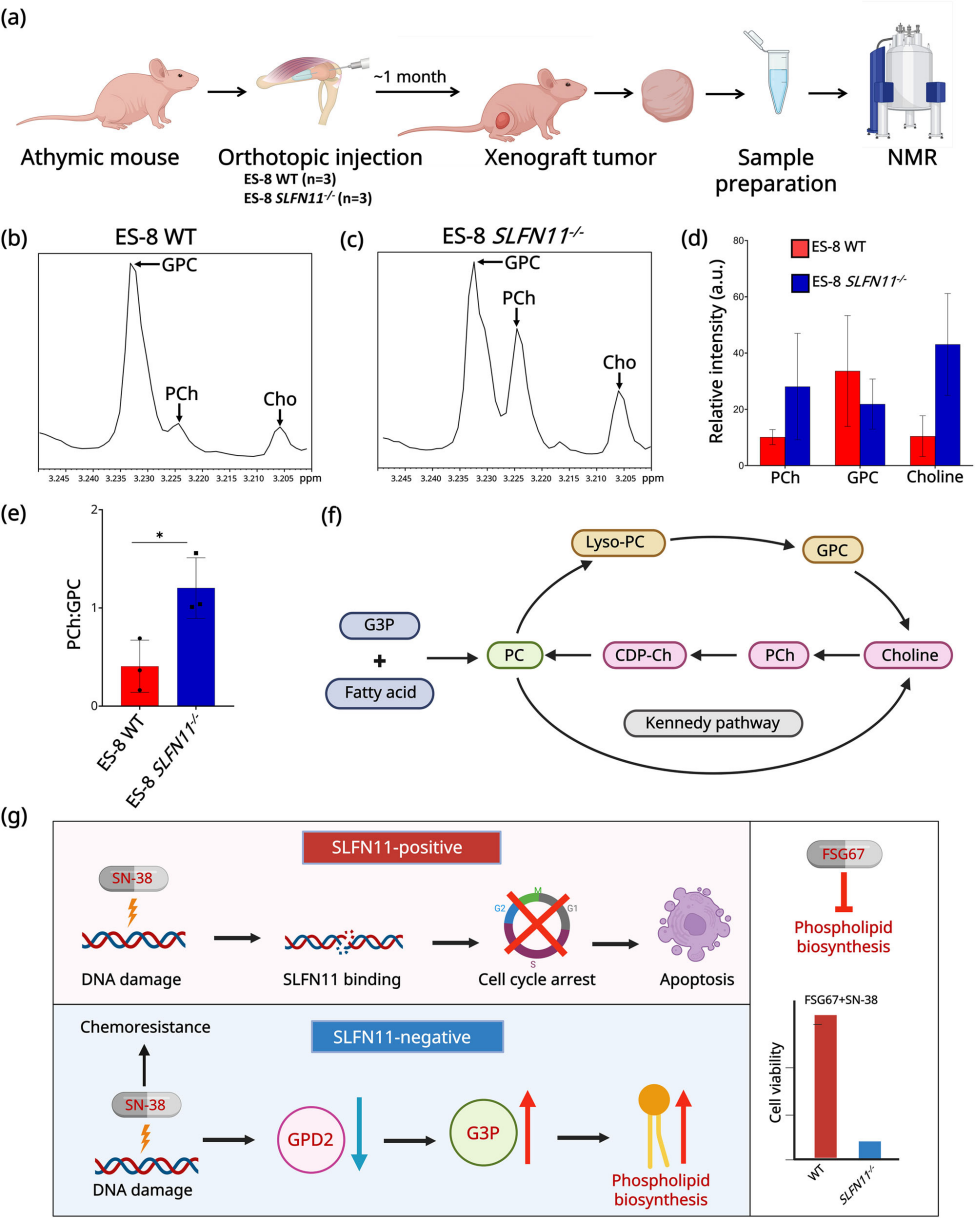
Ex vivo ¹H NMR profiling reveals altered choline metabolism in *SLFN11*^*−/−*^ EWS tumors. **a** Schematic of ex vivo ¹H NMR (600 MHz) analysis in athymic nude mice. EWS cells were implanted orthotopically, and excised tumors were extracted for metabolic profiling by ^1^H NMR. Representative ¹H NMR spectra of choline metabolite obtained from water-soluble tumor extract from ES-8 WT (**b**) and *SLFN11*^*−/−*^ (**c**) xenografts. Spectra are expanded to display signals from 3.20 to 3.25 ppm. Metabolites involved in choline metabolism including GPC (3.235 ppm), (PCh, 3.226 ppm), and free choline (3.208 ppm), highlighted with arrows. **d** Bar graph showing quantification of GPC, PCh, and total choline levels in WT and *SLFN11*^−/−^ ES-8 tumor xenografts. Data represent mean ± SD (*n* = 3). **e** Quantification of PCh/GPC ratio in ES-8 WT and *SLFN11*^*−/−*^ xenograft tumors. Data represent mean ± SD (*n* = 3). Statistical significance determined using paired two-tailed *t*-test (*p < 0.05). SD, standard deviation; *n* number of replicates. **f** Schematic illustrating the Kennedy pathway (pink) involved in choline metabolism, where choline is converted to PC via PCh and CDP-Ch. An alternate route involving G3P and fatty acids (blue) also contributes to PC generation. GPC and Lyso-PC degradation branches are shown in beige. PC phosphatidylcholine; CDP-Ch CDP-choline; PCh phosphocholine; GPC glycerophosphocholine; G3P glycerol-3-phosphate. **g** Schematic summary of *SLFN11*-loss-induced metabolic rewiring in EWS.

To determine whether the lipid metabolic reprogramming observed in vitro is reflected in vivo, we analyzed xenografted tumors derived from ES-8 WT and *SLFN11*^*−/−*^ cells. Nuclear magnetic resonance (NMR) spectroscopy was performed on metabolites extracted from these tumors in athymic nude mice, as outlined in Fig. 6a. [42, 58, 59]. NMR enables quantification of water-soluble metabolites, allowing evaluation of GPL metabolic alterations in tumor extracts [59]. Figure 6b and Fig. 6c show representative ¹H NMR spectra of tumor extracts of ES-8 WT and ES-8 *SLFN11*^*−/−*^ xenografts, respectively. Data averaged from three tumors per group revealed a consistent increase in phosphocholine (PCh) (*p* = 0.18) and total choline (Choline) (*p* = 0.04) levels in the ES-8 *SLFN11*^*−/−*^ group compared to ES-8 WT tumors (Fig. 6d). In contrast, Glycerophosphocholine (GPC) levels exhibited a downward trend (*p* = 0.39) in ES-8 *SLFN11*^*−/−*^ tumors (Fig. 6d). However, none of these changes reached statistical significance, likely reflecting variability across tumors and the challenges of resolving closely spaced resonances in the choline spectral region by NMR. Notably, the PCh/GPC ratio was significantly (~2.96-fold) elevated in the *SLFN11*^*−/−*^ group (*p* = 0.03) (Fig. 6e). For the comparison of PCh:GPC in ES-8 xenograft tumors (*SLFN11* knockout vs. WT) (Fig. 6e), a one-sided paired t-test with n = 3 per group provides 88.1% power to detect a true difference of ≥0.8 with standard deviation (SD) = 0.3 and 5% type I error (false positive) probability, indicating that the sample size was adequate for this analysis. In the Kennedy pathway, PCh contributes to GPL synthesis, while GPC results from GPL breakdown, making the PCh/GPC ratio indicative of GPL turnover (Fig. 6f). This shift toward higher PCh/GPC ratio in *SLFN11*^−/−^ tumors indicates enhanced choline-driven GPL biosynthesis in *SLFN11*^*−/−*^ xenografts.

Together, our findings show that *SLFN11* loss causes metabolic changes in EWS, including *GPD2* downregulation, G3P accumulation, and a higher PCh:GPC ratio. The elevated PCh:GPC ratio has been shown to correlate with tumor aggressiveness and treatment response [60, 61]. Hattingen et al. demonstrated that an elevated PCh:GPC ratio in recurrent glioblastoma was associated with shorter overall survival and resistance to antiangiogenic therapy, supporting its potential as a predictive biomarker [60]. These changes suggest increased GPL biosynthesis in *SLFN11*^−/−^ cells. This creates a metabolic vulnerability that can be targeted by combining DNA-damaging agents with lipid biosynthesis inhibitors. The proposed mechanism of metabolic reprogramming due to *SLFN11* loss is depicted schematically in Fig. 6g.

## Discussion

SLFN11 is a critical modulator of cancer cell sensitivity to DDAs [11, 13, 14, 16, 17, 62]. In response to DNA damage, *SLFN11* is recruited to replication forks via RPA1 [14, 17, 62] and interacts with the MCM3 [17, 62]. This interaction promotes chromatin opening and induces irreversible replication arrest without disrupting the loading of CDC45 [17, 62] or PCNA [62]. This mechanism contrasts with ATR-mediated fork slowing and allows SLFN11 to exert a dominant cytotoxic effect under genotoxic stress [63, 64]. Notably, SLFN11 functions by degrading CDT1 through DDB1–CUL4CDT2 E3 ubiquitin ligase complex, thereby blocking replication reactivation after damage [14]. Expression of *SLFN11* strongly correlates with responsiveness to a broad spectrum of DDAs, including topoisomerase I/II inhibitors, alkylating agents, DNA synthesis inhibitors, and PARP inhibitors, across multiple cancer types [4, 17]. Despite its therapeutic relevance, *SLFN11* is frequently inactivated, most commonly by promoter hypermethylation or histone modifications [13, 14, 17, 62]. This loss leads to chemoresistance in approximately 50% of cancer cell lines [13, 14]. Elevated *SLFN11* expression is associated with improved tumor-free survival in EWS, underscoring its clinical relevance [11]. Consistently, in our analysis of ESCLA, high *SLFN11* levels were strongly associated with improved overall and event-free patient survival. These findings support an association between *SLFN11* expression and improved clinical outcome and drug sensitivity in EWS. Additionally, DepMap dependency scores showed that *SLFN11* loss does not impair baseline viability in EWS cell lines, while transcriptomic profiles highlight that *SLFN11* expression provides a selective advantage under DDA treatment. Although *SLFN11* is expressed in the majority of EWS tumors, a subset (~10%) lacks detectable expression [15, 65], which may contribute to reduced sensitivity to DDAs. This underscores the importance of identifying alternative therapeutic strategies or predictive markers for *SLFN11*-negative EWS.

EZH2, the catalytic subunit of the Polycomb Repressive Complex 2 (PRC2), plays a central role in promoting therapeutic resistance by silencing tumor suppressor genes through H3K27 trimethylation [66]. It is frequently overexpressed in drug-resistant cancers and has been shown to suppress *SLFN11* expression, thereby impairing DNA damage response and contributing to chemoresistance [18]. Beyond its epigenetic function, EZH2 also regulates metabolic reprogramming to support tumor progression [66, 67]. Recent studies have demonstrated that EZH2 regulates lipid biosynthesis, mitochondrial dynamics, and redox balance, enabling cancer cells to adapt to metabolic stress [66–68]. In glioblastoma and immune cells, EZH2 activity promotes lipid accumulation and suppresses anti-tumor immunity, while in other models it enhances fatty acid synthesis to sustain proliferation and survival [68]. Our observations suggest that EZH2-mediated silencing of *SLFN11* may not only impair the DNA damage response but also facilitate the metabolic adaptations, including downregulation of *GPD2* and enhanced GPL biosynthesis. Such dual effects likely enhance tumor cell survival under genotoxic stress. Notably, EZH2 inhibition, by restoring SLFN11 expression and reversing lipid remodeling, may counteract these metabolic adaptations and help overcome therapy resistance.

In addition to these epigenetic effects, *SLFN11* loss may contribute to broader metabolic adaptations. The absence of *SLFN11* could alter the balance between energy production and biosynthetic demands or promote shifts in nutrient utilization that enable tumor cells to adapt under stress. Such reprogramming would allow cancer cells to couple impaired DNA damage response with enhanced metabolic flexibility, reinforcing their survival under genotoxic stress. Prior studies have leveraged metabolic characterization to uncover dependencies in oncogenic signaling and drug resistance across cancer types [35, 36, 53, 69–73]. Garrett *et al*. employed comprehensive metabolic profiling to characterize distinct metabolic programs across medulloblastoma subgroups, including alterations in purine and amino acid metabolism [72]. Stable isotope tracing has further showed nutrient utilization in both in vitro and in vivo models [35]. DeBerardinis and colleagues demonstrated that mitochondrial respiration sustains tricarboxylic acid (TCA) cycle activity and supports anabolic growth in non-small cell lung cancer (NSCLC), establishing mitochondrial glucose oxidation as a key feature of tumor metabolism [35, 53]. Additional studies from this group expanded on these insights by identifying a metabolic switch from de novo purine biosynthesis to salvage pathways under electron transport chain inhibition, driven by redox imbalance and augmented by purine nucleobase uptake in NSCLC [71]. In a parallel study, the same group showed that glutamine fuels TCA cycle intermediates via both oxidative and reductive pathways in ccRCC models [73]. Formate overflow from serine catabolism has been shown to promote tumor invasiveness [69, 70]. Acetate utilization, in turn, supports lipid synthesis as a compensatory adaptation to metabolic stress [36].

Building on these insights, our integrated transcriptomic and metabolomic profiling of *SLFN11*^*−/−*^ EWS models revealed a critical link between *SLFN11* loss-mediated chemoresistance and metabolic reprogramming, uncovering distinct vulnerabilities that may be exploited to overcome therapeutic resistance. A consistent feature was the downregulation of mitochondrial *GPD2*, a key component of the ETC and oxidative phosphorylation [22], leading to impaired mitochondrial function and cytosolic accumulation of G3P, a precursor for de novo GPL biosynthesis [51]. Yao et al. recently showed that in kidney cancer, loss of GPD2 uncouples the glycerol-3-phosphate shuttle. As a result, cells shift their metabolism from mitochondrial energy production to lipid synthesis, which helps tumors grow and maintain redox balance [22]. In glioblastoma, *GPD1* has been implicated in chemoresistance through its role in regulating GPL metabolism. Knockout of *GPD1* sensitizes glioblastoma cells to temozolomide, highlighting its contribution to therapy resistance [74]. Our LC-MS study with U-^13^C acetate tracing showed that elevated G3P levels drive the synthesis of key GPL, including PE, PC, PG, and PI, alongside increased expression of *AGPAT4*, a critical gene in GPL biosynthesis [63, 64]. While LC-MS/MS provides high sensitivity and broad metabolite coverage, analytical factors such as ionization efficiency [75], matrix effects [75], minor shifts in chromatographic separation or retention time [75], and isobaric or adduct interference[76] can influence metabolite detection and quantification [75, 76]. However, the consistent trends across replicates and correlation with transcriptomic data support the robustness of our findings. Multiple studies have shown that GPL synthesis rates are elevated during oncogenesis and tumor progression [77–81], including in lung [79], breast [78, 80], colorectal [81], bladder, and renal cancers [82] compared to normal tissue. Lesko et al. showed that lung cancer exhibits elevated de novo biosynthesis and turnover of phosphatidylethanolamine, indicating enhanced GPL metabolism in tumors [79]. Increased GPL biosynthesis also generates signaling lipids such as DAG and PA, which activate pro-survival pathways like mTOR [83]. *SLFN11* has been reported to suppress mTOR-driven tumorigenesis, suggesting that its loss may facilitate activation of lipid-sensitive survival signaling [84]. In addition to their structural role, GPL regulates oncogenic signaling, redox homeostasis, ferroptosis susceptibility, and therapeutic resistance [50, 77]. Increased GPL biosynthesis supports membrane remodeling [85], promotes proliferation [86], and limits lipid peroxidation [87], helping tumor cells withstand metabolic and therapeutic stress [50, 77]. Our observation of enhanced GPL biosynthesis in *SLFN11*^*−/−*^ EWS cells points to a potential role for lipid remodeling in sustaining chemoresistance within this population. We also observed shift toward elevated monounsaturated fatty acid synthesis, which can alter membrane fluidity. Such remodeling of lipid composition has been associated with enhanced tumor growth and aggressiveness [54–56]. Consistent with this metabolic shift, our in vivo metabolic analysis by NMR revealed an elevated PCh/GPC ratio in *SLFN11*^*−/−*^ EWS tumors. A high PCh to GPC ratio reflects elevated PC biosynthesis through Kennedy pathway and has been associated with increased tumor aggressiveness, cell proliferation, and therapeutic resistance across multiple cancers [42, 59, 61, 88]. NMR spectroscopy offers a complementary and quantitative approach to metabolic profiling but is limited by lower sensitivity, spectral overlap, narrower dynamic range, and longer acquisition times compared to LC-MS/MS [89]. However, the elevated PCh/GPC ratio observed here is consistent with established markers of PC biosynthesis and tumor aggressiveness. The elevated PCh/GPC ratio may serve as a functional biomarker of chemoresistance in EWS. Targeting de novo lipogenesis has been shown to overcome chemoresistance in several cancers [90]. Inhibitors of key lipogenic enzymes such as FASN, ACC, and SCD1 enhance drug sensitivity in models of pancreatic, ovarian, prostate, breast, and liver cancer, highlighting lipid biosynthesis as a promising therapeutic target [90]. Targeting GPAT1 with FSG67 has been shown to impair lipid synthesis and suppress tumor growth driven by GPD2 loss in kidney cancer, highlighting a metabolic vulnerability that can be therapeutically exploited [22].

While the precise mechanisms underlying the metabolic consequences of *SLFN11* loss remain to be defined, previous studies provide a framework for possible interpretation. SLFN11 has been shown to impair ribosome biogenesis by inhibiting rRNA synthesis and RNA polymerase I activity, thereby promoting TP53 independent apoptosis [91]. In this context, the absence of *SLFN11* may relieve this checkpoint, allowing sustained protein synthesis and activation of pro-growth pathways. Consistent with this, *SLFN11* downregulation has been associated with enhanced mTORC1 signaling [84], which can activate SREBP1 [92, 93], a key transcriptional regulator of fatty acid and phospholipid biosynthesis, to support membrane expansion and anabolic growth [93]. In line with this possibility, our findings show that *SLFN11* deficient EWS cells exhibit GPD2 downregulation with increased phospholipid biosynthesis, consistent with a metabolic shift from oxidative metabolism toward lipid-based anabolism. Although we did not directly examine ribosome biogenesis, it is plausible that loss of *SLFN11* sustains mTORC1 SREBP1 driven lipogenic reprogramming to promote metabolic adaptation and chemoresistance in EWS. Another plausible mechanism linking *SLFN11* loss to GPD2 suppression and lipid remodeling may involve shared epigenetic regulation, since *SLFN11* is frequently silenced by EZH2 mediated H3K27 trimethylation and promoter methylation [18], and similar chromatin repression may extend to genes involved in mitochondrial metabolism. A further possibility is that *SLFN11* loss enables activation of transcriptional programs such as SREBP1, MYC, or HIF1α that favor anabolic growth and suppress oxidative pathways [94]. GPD2 repression may also reflect mitochondrial stress signaling, as impaired mitochondrial function can activate retrograde pathways that reprogram nuclear metabolism, including lipid biosynthesis, to support adaptation [95]. Together, these scenarios outline plausible routes through which *SLFN11* deficiency could drive mitochondrial and metabolic rewiring to promote lipid biosynthesis and chemoresistance in EWS, providing a framework for future mechanistic investigation.

Loss of *SLFN11* expression in EWS reduces the effectiveness of DDAs, limiting therapeutic response and contributing to treatment resistance [11]. Previous studies have shown that *SLFN11*-expressing tumors respond robustly to SN-38 and other DDAs, whereas *SLFN11*^*−/−*^ EWS shows chemoresistance, presenting a major therapeutic challenge [4, 15]. Our findings suggest that *SLFN11* loss not only contributes to chemoresistance but also drives metabolic adaptations that support survival under genotoxic stress. We hypothesized that dual targeting of DNA damage and lipid biosynthesis pathways could counteract chemoresistance in *SLFN11*^*−/−*^ EWS. To assess the combined effect of both drugs, we employed BRAID modeling, which quantifies interaction strength through the kappa (κ) metric [38, 39]. Combination treatment with SN-38 and FSG67 significantly enhanced cell death in *SLFN11*^*−/−*^ cells, demonstrating a modest synergistic effect in case of ES-8 *SLFN11*^*−/−*^ (κ = 0.889) and SK ES-1 *SLFN11*^*−/−*^ (κ = 0.990). However, EW-8 *SLFN11*^*−/−*^ cell line showed a weak synergistic response (κ=0.221). Although the interaction did not reach statistical significance for either SK ES-1 or EW-8, the direction and magnitude of κ values were consistent with the underlying *SLFN11*-dependent response pattern. The variation in κ values across cell lines mirrors the underlying *SLFN11*-dependent response to SN-38. The smaller κ magnitude in EW-8 *SLFN11*^*−/−*^ cells is consistent with the weaker *SLFN11* effect on SN-38 sensitivity in this model [15]. This is reflected by a modest IAE_50_ difference between WT and *SLFN11*^*−/−*^ cells, in line with previously reported 0.59-fold lower baseline *SLFN11* expression in EW-8 compared to ES-8 cell line [15]. In contrast, no such synergy was observed in *SLFN11*-expressing WT cells. The modest synergy observed in *SLFN11*^*−/−*^ cells reflects the complexity of GPL metabolic networks, which may not be fully suppressed by single-agent inhibition. Combining GPL-targeting drugs with agents that converge on complementary pathways such as SREBP or mTOR signaling may enhance efficacy and broaden translational potential.

Our findings identify GPL metabolism as a candidate vulnerability in *SLFN11*-deficient EWS. Pharmacologic inhibition of lipid biosynthetic enzymes, as shown by our proof-of-concept data with FSG67, may overcome chemoresistance and could be combined with topoisomerase inhibitors in resistant EWS tumors. Translational progress will require GPL-targeting agents with pediatric safety profiles, biomarkers such as lipidomic signatures or GPD2 expression for patient selection, and validation in models that capture tumor-microenvironment and immune interactions. Addressing these challenges will be critical for advancing GPL metabolism inhibitors from preclinical studies to clinical application in EWS.

In summary, these findings reveal a shift towards GPL biosynthesis associated with *SLFN11* knockout in EWS that contributes to therapeutic resistance and exposes metabolic vulnerability. Co-targeting DNA damage and lipid biosynthesis pathways elicits a synergistic anti-tumor effect and offers a promising strategy to overcome DDA resistance in EWS.

### Limitations of the study

While our study offers important insights into how *SLFN11* loss drives metabolic adaptation and therapy resistance, several limitations should be acknowledged. In vivo validation was restricted to a single xenograft model (ES-8), which may not fully represent the metabolic heterogeneity across EWS tumors. Additionally, while transcriptomic and metabolomic data were integrated across multiple cell lines, the functional consequences of *GPD2* suppression and lipid remodeling were not evaluated using genetic rescue or knockdown models. Moreover, stable isotope tracing was performed under in vitro conditions, which may not fully mimic the metabolic constraints present in the tumor microenvironment. While BRAID analysis revealed synergy between SN-38 and FSG67 in *SLFN11*^*−/−*^ cells, the overall efficacy of this combination was modest, suggesting a need for further optimization or exploring alternative targets.

Another important limitation is that the regulatory mechanism connecting *SLFN11* loss with GPD2 suppression and lipid remodeling remains untested. Although we outlined several plausible explanations in the discussion, including shared epigenetic regulation, transcriptional reprogramming, and mitochondrial stress signaling that can reprogram nuclear gene expression, these possibilities remain speculative. Future studies using chromatin profiling, transcription factor activity assays, and genetic rescue or knockdown approaches will be essential to validate these hypotheses.

Upregulated lipid biosynthesis may also disrupt immune surveillance by exhausting CD8⁺ T cells, impairing NK cell function, and sustaining immunosuppressive Tregs and myeloid cells [96]. Although our study did not evaluate immune consequences directly, these findings suggest that GPL-driven immune dysfunction may also impact immunotherapy, and combining lipid biosynthesis inhibition, as demonstrated in our study, with immunotherapy could offer synergistic benefit.

## Supplementary information

Supplementary Information

Supplementary Information Original Western Blots

## Data Availability

All data reported in this study are available from the lead contact upon request.

## Materials availability

No unique reagents were generated in this study.
