# Supplementary material for: Targeting glycerophospholipid biosynthesis overcomes chemoresistance driven by SLFN11 loss in Ewing sarcoma
Source: Cell Death Dis. 2026 Jan 31;17(1):190. doi: 10.1038/s41419-026-08432-7 (PMC12877146; doi:10.1038/s41419-026-08432-7)
Supplement: Supplementary file 1 — Supplementary Information [file 41419_2026_8432_MOESM1_ESM.docx]

**Supplementary Information**

**Targeting glycerophospholipid biosynthesis overcomes chemoresistance driven by SLFN11 loss in Ewing sarcoma**

Kasturee Chakraborty^1^, Ritambhar Burman^1^, Saharsh Satheesh^1^, Matthew Kieffer^2^, Chandni Karuhatty^1^, Zuo-Fei Yuan^3^, Haiyan Tan^3^, Ankhbayar Lkhagva^3^, Anthony A High^3^, Xusheng Wang^4,5^, Alaa Refaat^6^, Nathaniel R. Twarog^6^, Weixing Zhang^7^, Yaxu Wang^8^, Yiping Fan^9^, Qian Li^10^, M Madan Babu^8^, Anang A Shelat^6^, Elizabeth Stewart^11^, Michael A Dyer^2^, and Puneet Bagga^1*^

^1^Department of Radiology, St. Jude Children's Research Hospital, Memphis, TN

^2^Department of Developmental Neurobiology, St. Jude Children's Research Hospital, Memphis, TN

^3^Center for Proteomics and Metabolomics, St. Jude Children's Research Hospital, Memphis, TN

^4^Department of Neurology, University of Tennessee Health Science Center, Memphis, TN

^5^Department of Genetics, Genomics and Informatics, University of Tennessee Health Science Center, Memphis, TN

^6^Department of Chemical Biology and Therapeutics, St. Jude Children's Research Hospital, Memphis, TN

^7^Department of Structural Biology, St. Jude Children's Research Hospital, Memphis, TN

^8^Center of Excellence for Data-Driven Discovery, Department of Structural Biology, St. Jude Children's Research Hospital, Memphis, TN

^9^Center for Applied Bioinformatics, St. Jude Children's Research Hospital, Memphis, TN

^10^Department of Biostatistics, St. Jude Children's Research Hospital, Memphis, TN

^11^Department of Oncology, St. Jude Children's Research Hospital, Memphis, TN

**Abbreviations:**

EWS, ewing sarcoma; DDA, DNA-damaging agent; *SLFN11*, schlafen family member 11; *GPD2*, glycerol-3-phosphate dehydrogenase 2; GPL, glycerophospholipid; EZH2, enhancer of zeste homolog 2; G3P, glycerol-3-phosphate; CCLE, Cancer Cell Line Encyclopedia; DepMap, cancer dependency map; CRISPR, clustered regularly interspaced short palindromic repeats; RNAi,  RNA interference; FDR, false discovery rate; TCGA, The Cancer Genome Atlas Program; ESCLA, Ewing Sarcoma Cell Line Atlas; *EWSR1*, ewing sarcoma breakpoint region 1; ETS, erythroblast transformation specific; RNA-seq, RNA sequencing; PCA, principal component analysis; PC1, first principal component; Log_2_FC, Log_2_ fold change; WT, wild type; *SLFN11^-/-^*; *SLFN11* knock out; Pearson R, Pearson correlation coefficient; G3PS, glycerol-3-phosphate shuttle; DHAP, dihydroxyacetone phosphate; ETC, electron transport chain; LC/MS, Liquid chromatography–mass spectrometry; PE, phosphatidylethanolamine; PC, phosphatidylcholine; PI, phosphatidylinositol; PG, phosphatidylglycerol; PA, phosphatidic acid; DAG, diacylglycerol; *AGPAT4*, 1-Acylglycerol-3-Phosphate O-Acyltransferase 4; MUFA, monounsaturated fatty acid; *GPAT1*, glycerol-3-phosphate acyltransferase 1; BRAID, Bivariate Response to Additive Interacting Doses; *GPAT1*, glycerol-3-phosphate acyltransferase 1; IAE, Index of Achievable Efficacy; EC_50_, half maximal effective concentration; ^1^H NMR, proton nuclear magnetic resonance; PCh, phosphocholine; GPC, glycerophosphocholine; RPA1, replication protein A; MCM3, minichromosome maintenance complex component 3; CDC45, cell division cycle 45; PCNA, proliferating cell nuclear antigen; ATR, Ataxia Telangiectasia and Rad3-related; CDT, chromatin licensing and DNA replication factor 1; DDB1, damage-binding protein 1; CUL4, cullin 4; PARP, poly (ADP-ribose) polymerase; NSCLC, non-small cell lung cancer; ccRCC, clear cell renal cell carcinoma; mTOR, mammalian target of rapamycin; FASN, fatty acid synthase; ACC, acetyl-CoA carboxylase; SCD1, stearoyl-CoA desaturase 1.

**Table S1.** Summary of EWS cell lines used in this study.

| **Cell Line** | **Source** | **Age (years)** | **Sex** | **Primary Site** | **Metastatic Site** | **Translocation Type** |
| --- | --- | --- | --- | --- | --- | --- |
| ES-8 | SJCRH | 10 | M | Left proximal humerus | Femur | EWSR1 Exon 7 to FLI1 Exon 5 (Type II) |
| SK ES-1 | ATCC | 18 | M | Bone | - | EWSR1 Exon 7 to FLI1 Exon 5 (Type II) |
| EW-8 | SJCRH | 17 | M | Abdominal mass | - | EWSR1 Exon 7 to FLI1 Exon 6 (Type I) |
| RD ES-1 | ATCC | 19 | M | Bone | - | EWSR1 Exon 7 to FLI1 Exon 5 (Type II) |
| CADO ES-1 | Center for Adult Diseases Osaka | 19 | F | - | Lung | EWSR1-ERG |

**
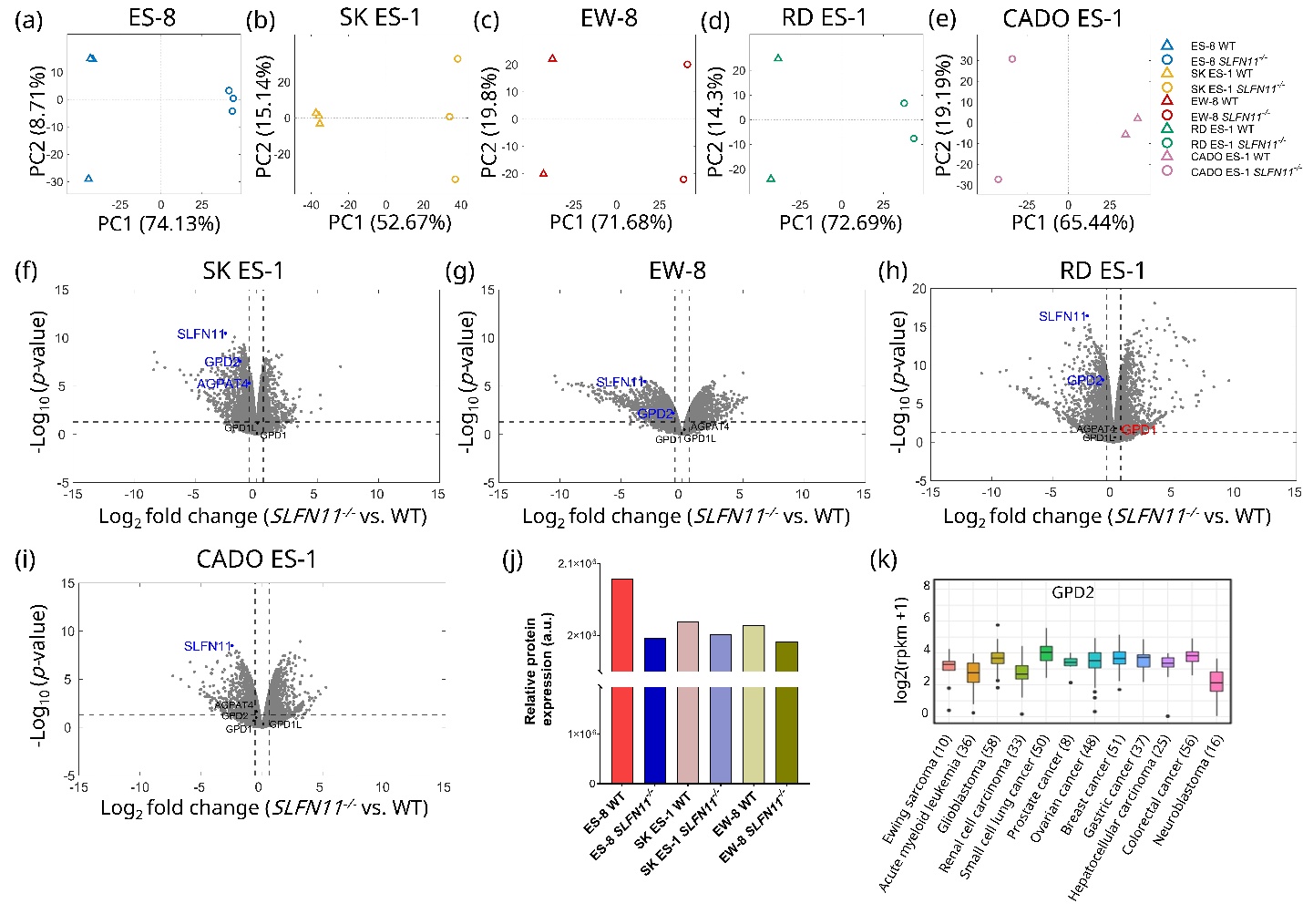
**

**Figure S1. *SLFN11* knockout is associated with suppression of *GPD2* across EWS cell lines. (a-e)** PCA of RNA-seq data from WT vs. *SLFN11^-/-^* demonstrating segregation along PC1 in (a) ES-8, (b) SK ES-1, (c) EW-8, (d) RD ES-1 and (e) CADO ES-1 cell lines. Each point represents a biological replicate, with triangles indicating WT and circles indicating *SLFN11^-/-^* samples. Color coding for each cell line and genotype is provided alongside the figure. **(f-i)** Volcano plots showing differentially expressed genes in *SLFN11*^-/-^ versus WT cells from RNA-seq analysis. Red dots indicate significantly upregulated genes involved, while blue dots represent significantly downregulated genes in G3PS in **(f)** SK ES-1, **(g)** EW-8, **(h)** RD-ES-1, and **(i)** CADO-ES-1 cell lines. **(j)** Densitometric analysis of GPD2 protein expression from Western blot in WT and *SLFN11*⁻/⁻ EWS cell lines ES-8, SK ES-1, and EW-8. Band intensities from Western blot analysis were normalized to β-actin. **(k)** Expression of *GPD2* across multiple cancer subtypes from the CCLE dataset. Box plots represent log₂-transformed RPKM values (log₂[RPKM + 1]). Sample numbers (n) are indicated in parentheses.

**
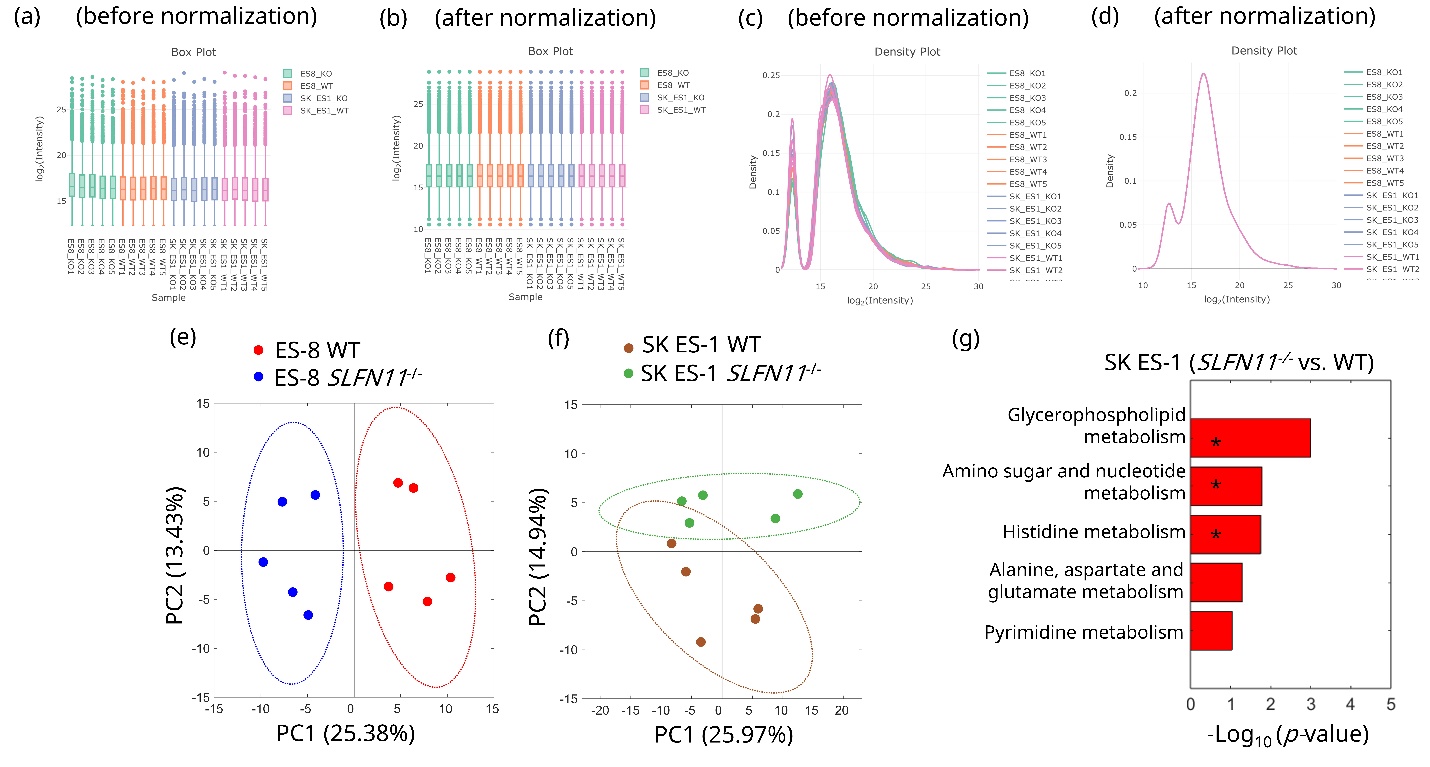
**

**Figure S2. Data normalization and PCA reveal metabolic differences between *SLFN11^-/-^* and WT EWS cell lines (a-b)** Box plots showing log₂-transformed metabolite intensity distributions across ES-8 WT and ES-8 *SLFN11^-/-^*, SK ES-1 WT and SK ES-1 *SLFN11^-/-^* biological replicates, **(a)** before and **(b)** after quantile normalization. Each box represents one sample; colors indicate genotype and cell line**. (c-d)** Density plots showing log₂-transformed metabolite intensity distributions across ES-8 WT and ES-8 *SLFN11^-/-^*, SK ES-1 WT and SK ES-1 *SLFN11^-/-^* biological replicates, **(c)** before and **(d)** after quantile normalization. Each curve represents one sample. Color coding reflects sample identity and genotype **(e-f)** PCA of metabolomic profiling from **(e)** ES-8 (*SLFN11^-/-^* vs. WT) and **(f)** SK ES-1 (*SLFN11^-/-^* vs. WT) cell lines demonstrating segregation along PC1. Each dot represents an individual replicate. Color coding indicates genotype and cell line**. (g)** MSEA-based pathway enrichment analysis identifying upregulated metabolic pathways in SK ES-1 *SLFN11^-/-^* cell line. Pathways with –log_10_ (FDR) ≥ 0.5 were considered enriched and ranked by significance. Asterisks mark statistically significant pathways.

| **Metabolite** | **HMDB ID** | **FDR** | **Log2Fold (ES-8 SLFN11^-/-^vs. ES-8 WT)** |
| --- | --- | --- | --- |
| Dihydroorotate | HMDB0003349 | 2.43E-03 | 0.51 |
| Uridine 5'-Diphosphate | HMDB0000295 | 1.52E-01 | 0.54 |
| Cytidine Monophosphate | HMDB0000095 | 7.33E-03 | 0.7 |
| Adenosine 5'-Triphosphate | HMDB0000538 | 1.69E-01 | 0.77 |
| D-Ribose 5-Phosphate | HMDB0001548 | 2.29E-03 | 0.91 |
| Heptadecanoic acid | HMDB0002259 | 1.18E-02 | 1.05 |
| Xanthine | HMDB0000292 | 9.61E-09 | 1.07 |
| Folate | HMDB0000121 | 9.98E-05 | 1.12 |
| Vaccenic Acid | HMDB0240219 | 1.25E-04 | 1.2 |
| Uridine 5'-Triphosphate | HMDB0000285 | 4.78E-02 | 1.29 |
| Serine | HMDB0000187 | 1.61E-01 | 1.31 |
| Nervonic Acid | HMDB0002368 | 1.31E-04 | 1.4 |
| Phosphoenolpyruvate | HMDB0000263 | 9.31E-02 | 1.61 |
| Uridine Monophosphate | HMDB0000288 | 6.61E-09 | 2.22 |
| **Glycerol 3-Phosphate** | **HMDB0000126** | **1.43E-06** | **2.28** |
| Inosine | HMDB0000195 | 4.32E-07 | 3.37 |
| Citrate | HMDB0000094 | 6.80E-02 | 3.65 |
| **O-Phosphoethanolamine** | **HMDB0000224** | **9.07E-10** | **3.9** |
| Inosine 5'-Monophosphate | HMDB0000175 | 2.50E-04 | 4.01 |

**Table S2.** Selected metabolically important upregulated metabolites among the differentially regulated metabolites in ES-8 *SLFN11^-/-^* cell line compared to ES-8 WT.

**Table S3.** Selected metabolically important upregulated metabolites among the differentially regulated metabolites in SK ES-1 *SLFN11^-/-^* cell line compared to SK ES-1 WT.

| **Metabolite** | **HMDB ID** | **FDR** | **Log2Fold (SK ES-1 SLFN11^-/-^ vs. SK ES-1 WT)** |
| --- | --- | --- | --- |
| Cytidine Monophosphate | HMDB0000095 | 4.13E-03 | 0.8 |
| Uridine Monophosphate | HMDB0000288 | 1.29E-04 | 1.24 |
| **Glycerol 3-Phosphate** | **HMDB0000126** | **7.33E-04** | **1.5** |
| L-Glutamic Acid | HMDB0000148 | 3.07E-01 | 1.93 |
| **O-Phosphoethanolamine** | **HMDB0000224** | **1.15E-08** | **3.63** |

**Table S4.** Isotopologue distribution pattern of metabolites labeled with U-^13^C glucose in ES-8 cell line.

| **Labeling %** | **Isotopologue** | **ES-8 WT** | **ES-8 WT** | **ES-8 WT** | **ES-8 *SLFN11^-/-^*** | **ES-8 *SLFN11^-/-^*** | **ES-8 *SLFN11^-/-^*** |
| --- | --- | --- | --- | --- | --- | --- | --- |
| Pyruvate | M+3 | 99.86 | 99.94 | 100.19 | 99.09 | 89.72 | 98.16 |
| Lactate | M+3 | 87.68 | 90.69 | 90.48 | 91.14 | 91.18 | 91.27 |
| Citrate | M+2 | 32.57 | 30.29 | 42.51 | 32.96 | 34.85 | 34.44 |
| Glutamate | M+2 | 23.89 | 22.43 | 23.20 | 22.06 | 21.18 | 21.80 |
| Fumarate | M+2 | 23.15 | 21.71 | 19.81 | 25.12 | 25.92 | 24.39 |
| Malate | M+2 | 25.24 | 24.47 | 28.99 | 25.13 | 26.96 | 25.65 |
| Malate | M+3 | 21.09 | 23.41 | 22.95 | 22.36 | 25.42 | 23.10 |
| Fumarate | M+3 | 16.64 | 23.03 | 14.62 | 20.67 | 21.14 | 21.71 |
| G3P | M+3 | 35.34 | 51.64 | 39.74 | 89.67 | 92.66 | 92.59 |
| DHAP | M+3 | 90.29 | 92.20 | 89.54 | 92.64 | 93.98 | 95.30 |

**Table S5.** Isotopologue distribution pattern of metabolites labeled with U-^13^C glucose in SK ES-1 cell line.

| **Labeling %** | **Isotopologue** | **SK ES-1** **WT** | **SK ES-1** **WT** | **SK ES-1** **WT** | **SK ES-1** **SLFN11^-/-^** | **SK ES-1** **SLFN11^-/-^** | **SK ES-1** **SLFN11^-/-^** |
| --- | --- | --- | --- | --- | --- | --- | --- |
| Lactate | M+3 | 78.39 | 76.30 | 83.01 | 73.24 | 89.68 | 89.17 |
| Citrate | M+2 | 22.89 | 36.26 | 39.81 | 30.14 | 31.77 | 34.87 |
| Glutamate | M+2 | 23.73 | 21.50 | 23.16 | 21.14 | 21.83 | 22.17 |
| Fumarate | M+2 | 17.91 | 16.01 | 19.15 | 15.86 | 15.46 | 18.73 |
| Malate | M+2 | 20.37 | 17.93 | 18.94 | 16.61 | 17.78 | 18.83 |
| Malate | M+3 | 10.46 | 9.29 | 9.71 | 7.53 | 8.37 | 8.82 |
| Fumarate | M+3 | 4.57 | 3.99 | 4.68 | 3.26 | 3.23 | 5.21 |
| G3P | M+3 | 89.06 | 89.74 | 89.49 | 94.42 | 95.13 | 93.40 |
| DHAP | M+3 | 93.41 | 89.88 | 89.01 | 93.22 | 94.81 | 93.61 |

**Table S6.** Isotopologue distribution pattern of metabolites labeled with U-^13^C glucose in EW-8 cell line.

| **Labeling %** | **Isotopologue** | **EW-8 WT** | **EW-8 WT** | **EW-8 WT** | **EW-8 *SLFN11^-/-^*** | **EW-8 *SLFN11^-/-^*** | **EW-8 *SLFN11^-/-^*** |
| --- | --- | --- | --- | --- | --- | --- | --- |
| Pyruvate | M+3 | 90.14 | 91.57 | 91.58 | 92.34 | 91.48 | 92.27 |
| Lactate | M+3 | 90.60 | 91.41 | 90.81 | 91.28 | 92.55 | 92.30 |
| Citrate | M+2 | 35.92 | 47.18 | 50.53 | 39.68 | 46.63 | 48.25 |
| Glutamate | M+2 | 23.15 | 23.07 | 23.22 | 25.38 | 25.17 | 25.31 |
| Fumarate | M+2 | 20.22 | 22.06 | 21.16 | 22.13 | 24.08 | 23.45 |
| Malate | M+2 | 21.18 | 20.90 | 21.02 | 23.17 | 22.79 | 22.96 |
| Malate | M+3 | 13.14 | 12.90 | 13.09 | 15.98 | 16.12 | 16.12 |
| Fumarate | M+3 | 11.72 | 11.75 | 11.49 | 14.48 | 14.47 | 15.27 |
| G3P | M+3 | 86.73 | 85.66 | 84.6 | 91.38 | 91.56 | 91.64 |
| DHAP | M+3 | 94.59 | 94.32 | 94.33 | 96.24 | 96.32 | 96.06 |

**Table S7.** Up and downregulated pathways in ES-8 *SLFN11^-/-^* cell line compared to ES-8 WT.

| **Upregulated pathway** | **Overlapped HMDB IDs** | **Overlapped metabolites** | **# metabolites overlapped** | **# metabolites in the pathway** | **p-value** |
| --- | --- | --- | --- | --- | --- |
| Pyrimidine metabolism | HMDB0000295 | Uridine 5'-diphosphate | 7 | 39 | 1.01E-05 |
|  | HMDB0000285 | Uridine 5'-triphosphate |  |  |  |
|  | HMDB0000288 | Uridine 5'- monophosphate |  |  |  |
|  | HMDB0000095 | Cytidine monophosphate |  |  |  |
|  | HMDB0001202 | dCMP |  |  |  |
|  | HMDB0000528 | 4,5-Dihydroorotic acid |  |  |  |
|  | HMDB0000828 | Ureidosuccinic acid |  |  |  |
| Purine  metabolism | HMDB0001548 | D-Ribose 5-phosphate | 7 | 70 | 4.89E-04 |
|  | HMDB0000462 | Allantoin |  |  |  |
|  | HMDB0000538 | Adenosine triphosphate |  |  |  |
|  | HMDB0000175 | Inosinic acid |  |  |  |
|  | HMDB0000195 | Inosine |  |  |  |
|  | HMDB0000292 | Xanthine |  |  |  |
|  | HMDB0000299 | Xanthosine |  |  |  |
| One carbon pool by folate | HMDB0000121 | Folic acid | 4 | 26 | 1.86E-03 |
|  | HMDB0000187 | Serine |  |  |  |
|  | HMDB0000538 | Adenosine triphosphate |  |  |  |
|  | HMDB0000939 | S-Adenosylhomocysteine |  |  |  |
| Alanine, aspartate, and glutamate metabolism | HMDB0000094 | Citric acid | 3 | 28 | 2.02E-02 |
|  | HMDB0000828 | Ureidosuccinic acid |  |  |  |
|  | HMDB0001254 | Glucosamine 6-phosphate |  |  |  |
| Glycerophospholipid metabolism | HMDB0000126 | Glycerol 3-phosphate | 2 | 36 | 5.71e-2 |
|  | HMDB0000224 | O-Phosphoethanolamine |  |  |  |
| **Downregulated pathway** | **Overlapped HMDB IDs** | **Overlapped metabolites** | **# metabolites overlapped** | **# metabolites in the pathway** | **p-value** |
| Alanine, aspartate, and glutamate metabolism | HMDB0000812 | N-Acetyl-L-aspartic acid | 5 | 28 | 1.08E-04 |
|  | HMDB0006483 | D-Aspartic acid |  |  |  |
|  | HMDB0000168 | L-Asparagine |  |  |  |
|  | HMDB0000208 | Oxoglutaric acid |  |  |  |
|  | HMDB0000134 | Fumaric acid |  |  |  |
| Citrate cycle (TCA cycle) | HMDB0000156 | Malic acid | 4 | 20 | 3.67E-04 |
|  | HMDB0000134 | Fumaric acid |  |  |  |
|  | HMDB0000072 | cis-Aconitic acid |  |  |  |
|  | HMDB0000208 | Oxoglutaric acid |  |  |  |
| Pyruvate metabolism | HMDB0001066 | S-Lactoylglutathione | 3 | 23 | 7.76E-03 |
|  | HMDB0000156 | Malic acid |  |  |  |
|  | HMDB0000134 | Fumaric acid |  |  |  |
| Arginine biosynthesis | HMDB0000208 | Oxoglutaric acid | 2 | 14 | 2.60E-02 |
|  | HMDB0000134 | Fumaric acid |  |  |  |
| Histidine metabolism | HMDB0000033 | Carnosine | 2 | 16 | 3.35E-02 |
|  | HMDB0000001 | 1-Methylhistidine |  |  |  |

**Table S8.** Up and downregulated pathways in SK ES-1 *SLFN11^-/-^* cell line compared to SK ES-1 WT.

| **Upregulated pathway** | **Overlapped HMDB IDs** | **Overlapped metabolites** | **# metabolites overlapped** | **# metabolites in the pathway** | **p-value** |
| --- | --- | --- | --- | --- | --- |
| Glycerophospholipid metabolism | HMDB0000126 | Glycerol 3-phosphate | 3 | 36 | 9.30e-3 |
|  | HMDB0000224 | O-Phosphoethanolamine |  |  |  |
|  | HMDB0008834 | PE(14:0/20:1(11Z)) |  |  |  |
| Amino sugar and nucleotide sugar metabolism | HMDB0001254 | Glucosamine 6-phosphate | 3 | 42 | 1.63E-02 |
|  | HMDB0000290 | Uridine diphosphate-N-acetylglucosamine |  |  |  |
|  | HMDB0001163 | Guanosine diphosphate mannose |  |  |  |
| Histidine metabolism | HMDB0000033 | Carnosine | 2 | 16 | 1.77E-02 |
|  | HMDB0000148 | Glutamic acid |  |  |  |
| Alanine, aspartate and glutamate metabolism | HMDB0000148 | Glutamic acid | 2 | 28 | 5.09E-02 |
|  | HMDB0001254 | Glucosamine 6-phosphate |  |  |  |
| Pyrimidine metabolism | HMDB0000288 | Uridine 5'-monophosphate | 2 | 39 | 9.16E-02 |
|  | HMDB0000095 | Cytidine monophosphate |  |  |  |
| **Downregulated pathway** | **Overlapped HMDB IDs** | **Overlapped metabolites** | **# metabolites overlapped** | **# metabolites in the pathway** | **p-value** |
| Alanine, aspartate, and glutamate metabolism | HMDB0006483 | D-Aspartic acid | 5 | 28 | 3.91E-05 |
|  | HMDB0000828 | Ureidosuccinic acid |  |  |  |
|  | HMDB0000208 | Oxoglutaric acid |  |  |  |
|  | HMDB0000134 | Fumaric acid |  |  |  |
|  | HMDB0000254 | Succinic acid |  |  |  |
| Citrate cycle (TCA cycle) | HMDB0000156 | Malic acid | 4 | 20 | 1.64E-04 |
|  | HMDB0000254 | Succinic acid |  |  |  |
|  | HMDB0000134 | Fumaric acid |  |  |  |
|  | HMDB0000208 | Oxoglutaric acid |  |  |  |
| Purine metabolism | HMDB0001517 | AICAR | 6 | 70 | 4.19E-04 |
|  | HMDB0000133 | Guanosine |  |  |  |
|  | HMDB0000045 | Adenosine monophosphate |  |  |  |
|  | HMDB0000034 | Adenine |  |  |  |
|  | HMDB0000195 | Inosine |  |  |  |
|  | HMDB0000292 | Xanthine |  |  |  |
| One carbon pool by folate | HMDB0000121 | Folic acid | 4 | 26 | 4.77E-04 |
|  | HMDB0000187 | Serine |  |  |  |
|  | HMDB0000092 | Dimethylglycine |  |  |  |
|  | HMDB0000099 | L-Cystathionine |  |  |  |
| Arginine biosynthesis | HMDB0000208 | Oxoglutaric acid | 3 | 14 | 9.84E-04 |
|  | HMDB0000904 | Citrulline |  |  |  |
|  | HMDB0000134 | Fumaric acid |  |  |  |
| Glycine, serine, and threonine metabolism | HMDB0000092 | Dimethylglycine | 4 | 33 | 1.21E-03 |
|  | HMDB0001149 | 5-Aminolevulinic acid |  |  |  |
|  | HMDB0000099 | L-Cystathionine |  |  |  |
|  | HMDB0000187 | Serine |  |  |  |
| Pyruvate metabolism | HMDB0001066 | S-Lactoylglutathione | 3 | 23 | 4.37E-03 |
|  | HMDB0000156 | Malic acid |  |  |  |
|  | HMDB0000134 | Fumaric acid |  |  |  |
| D-Amino acid metabolism | HMDB0006483 | D-Aspartic acid | 2 | 15 | 2.02E-02 |
|  | HMDB0000187 | Serine |  |  |  |
| Butanoate metabolism | HMDB0000208 | Oxoglutaric acid | 2 | 15 | 2.02E-02 |
|  | HMDB0000254 | Succinic acid |  |  |  |

**Table S9.** Isotopologue distribution pattern of metabolites labeled with U^13^C acetate in ES-8 cell line.

| **Phospholipid species** | **Isotopologues** | **% Enrichment** | | | | ***p*-value**  **(ES-8 SLFN11-/- vs WT)** |
| --- | --- | --- | --- | --- | --- | --- |
|  |  | **ES-8 WT** | | **ES-8 *SLFN11^-/-^*** | |  |
|  |  | **Avg** | **SD** | **Avg** | **SD** |  |
| PE (34:1) | M+0 | 24.6 | 13.7 | 18.0 | 12.0 | 2.72E-03 |
|  | M+1 | 0.3 | 0.3 | 0.4 | 0.2 | 9.63E-01 |
|  | M+2 | 21.2 | 11.9 | 15.3 | 10.1 | 4.91E-02 |
|  | M+3 | 0.6 | 0.5 | 0.6 | 0.1 | 9.09E-01 |
|  | M+4 | 15.2 | 8.5 | 11.1 | 7.4 | 8.97E-02 |
|  | M+5 | 0.5 | 0.5 | 0.5 | 0.1 | 5.45E-01 |
|  | M+6 | 7.9 | 4.3 | 5.7 | 3.6 | 3.57E-02 |
|  | M+7 | 0.1 | 0.1 | 0.2 | 0.1 | 1.91E-01 |
|  | M+8 | 3.7 | 2.0 | 2.7 | 1.7 | 2.38E-02 |
|  | M+9 | 0.0 | 0.0 | 0.0 | 0.0 | 2.02E-01 |
|  | M+10 | 1.4 | 0.7 | 1.1 | 0.6 | 8.22E-03 |
|  | M+11 | 0.0 | 0.0 | 0.1 | 0.0 | 8.39E-01 |
|  | M+12 | 0.6 | 0.2 | 0.4 | 0.2 | 1.56E-03 |
| PC (34:1) | M+0 | 16.8 | 11.4 | 14.1 | 2.7 | 3.18E-03 |
|  | M+1 | 0.3 | 0.1 | 0.2 | 0.1 | 7.74E-01 |
|  | M+2 | 19.1 | 13.2 | 16.2 | 2.9 | 8.86E-04 |
|  | M+3 | 1.0 | 0.6 | 0.8 | 0.2 | 2.51E-01 |
|  | M+4 | 15.0 | 10.4 | 12.7 | 2.3 | 2.53E-04 |
|  | M+5 | 0.5 | 0.2 | 0.4 | 0.2 | 1.19E-01 |
|  | M+6 | 8.4 | 5.6 | 7.0 | 1.4 | 2.34E-03 |
|  | M+7 | 0.5 | 0.3 | 0.4 | 0.1 | 5.99E-02 |
|  | M+8 | 3.7 | 2.4 | 3.0 | 0.6 | 1.69E-03 |
|  | M+9 | 0.2 | 0.1 | 0.1 | 0.0 | 7.81E-02 |
|  | M+10 | 1.4 | 0.9 | 1.2 | 0.2 | 2.33E-03 |
|  | M+11 | 0.1 | 0.0 | 0.1 | 0.0 | 2.73E-02 |
|  | M+12 | 0.5 | 0.3 | 0.4 | 0.1 | 1.58E-03 |
| PG (34:1) | M+0 | 17.9 | 10.1 | 13.0 | 8.7 | 2.21E-09 |
|  | M+1 | 0.5 | 0.3 | 0.4 | 0.1 | 1.00E-01 |
|  | M+2 | 20.5 | 11.2 | 15.0 | 9.8 | 1.35E-07 |
|  | M+3 | 0.4 | 0.2 | 0.4 | 0.1 | 1.18E-01 |
|  | M+4 | 15.8 | 8.8 | 11.6 | 7.7 | 3.88E-01 |
|  | M+5 | 0.6 | 0.3 | 0.5 | 0.1 | 9.92E-01 |
|  | M+6 | 9.6 | 5.1 | 6.8 | 4.3 | 4.02E-05 |
|  | M+7 | 0.3 | 0.3 | 0.4 | 0.1 | 5.04E-01 |
|  | M+8 | 4.9 | 2.5 | 3.5 | 2.1 | 2.81E-08 |
|  | M+9 | 0.2 | 0.1 | 0.2 | 0.1 | 5.68E-01 |
|  | M+10 | 1.8 | 0.9 | 1.3 | 0.7 | 3.44E-08 |
|  | M+11 | 0.0 | 0.0 | 0.1 | 0.0 | 5.17E-02 |
|  | M+12 | 2.1 | 1.1 | 1.6 | 0.8 | 1.24E-02 |
| PI (34:1) | M+0 | 19.3 | 10.6 | 14.2 | 9.4 | 1.03E-02 |
|  | M+1 | 0.6 | 0.4 | 0.5 | 0.1 | 1.19E-01 |
|  | M+2 | 21.5 | 11.9 | 15.8 | 10.5 | 3.29E-03 |
|  | M+3 | 0.3 | 0.3 | 0.3 | 0.0 | 3.80E-01 |
|  | M+4 | 17.3 | 9.8 | 12.6 | 8.5 | 8.91E-03 |
|  | M+5 | 0.3 | 0.1 | 0.2 | 0.1 | 4.52E-02 |
|  | M+6 | 9.5 | 5.2 | 6.8 | 4.5 | 1.26E-02 |
|  | M+7 | 0.6 | 0.5 | 0.5 | 0.1 | 5.61E-01 |
|  | M+8 | 4.5 | 2.2 | 3.2 | 1.8 | 2.45E-03 |
|  | M+9 | 0.2 | 0.1 | 0.2 | 0.1 | 3.97E-01 |
|  | M+10 | 1.6 | 0.8 | 1.1 | 0.7 | 1.37E-03 |
|  | M+11 | 0.0 | 0.0 | 0.0 | 0.0 | 5.22E-01 |
|  | M+12 | 0.7 | 0.3 | 0.5 | 0.2 | 5.38E-02 |
| Oleate | M+0 | 84.1 | 2.8 | 75.3 | 4.6 | 1.19E-02 |
|  | M+1 | 0.1 | 0.2 | 0.3 | 0.2 | 3.59E-01 |
|  | M+2 | 9.4 | 1.7 | 8.8 | 1.9 | 6.74E-01 |
|  | M+3 | 0.0 | 0.0 | 0.0 | 0.0 | 3.47E-01 |
|  | M+4 | 4.5 | 0.9 | 8.1 | 1.5 | 3.34E-03 |
|  | M+5 | 0.0 | 0.0 | 0.0 | 0.0 | 3.47E-01 |
|  | M+6 | 1.4 | 0.3 | 4.7 | 0.9 | 1.20E-04 |
|  | M+7 | 0.0 | 0.0 | 0.0 | 0.0 | 4.22E-02 |
|  | M+8 | 0.4 | 0.1 | 1.9 | 0.4 | 3.04E-05 |
|  | M+9 | 0.0 | 0.0 | 0.0 | 0.0 | 3.47E-01 |
|  | M+10 | 0.1 | 0.0 | 0.6 | 0.1 | 3.89E-05 |
|  | M+11 | 0.0 | 0.0 | 0.0 | 0.0 | 1.41E-01 |
|  | M+12 | 0.0 | 0.0 | 0.2 | 0.0 | 1.03E-05 |
| Palmitate | M+0 | 98.5 | 0.4 | 98.6 | 0.2 | 4.25E-01 |
|  | M+1 | 0.8 | 0.1 | 0.6 | 0.1 | 2.40E-01 |
|  | M+2 | 0.1 | 0.2 | 0.0 | 0.0 | 1.00E-01 |
|  | M+3 | 0.1 | 0.0 | 0.1 | 0.0 | 8.67E-01 |
|  | M+4 | 0.3 | 0.2 | 0.3 | 0.1 | 7.80E-01 |
|  | M+5 | 0.0 | 0.0 | 0.0 | 0.0 | 4.21E-01 |
|  | M+6 | 0.1 | 0.1 | 0.2 | 0.0 | 2.25E-01 |
|  | M+7 | 0.0 | 0.0 | 0.0 | 0.0 | 5.56E-01 |
|  | M+8 | 0.0 | 0.0 | 0.1 | 0.0 | 5.53E-02 |
|  | M+9 | 0.0 | 0.0 | 0.0 | 0.0 | 3.47E-01 |
|  | M+10 | 0.0 | 0.0 | 0.0 | 0.0 | 3.67E-02 |
|  | M+11 | 0.0 | 0.0 | 0.0 | 0.0 | NA |
|  | M+12 | 0.0 | 0.0 | 0.0 | 0.0 | 3.47E-01 |

**
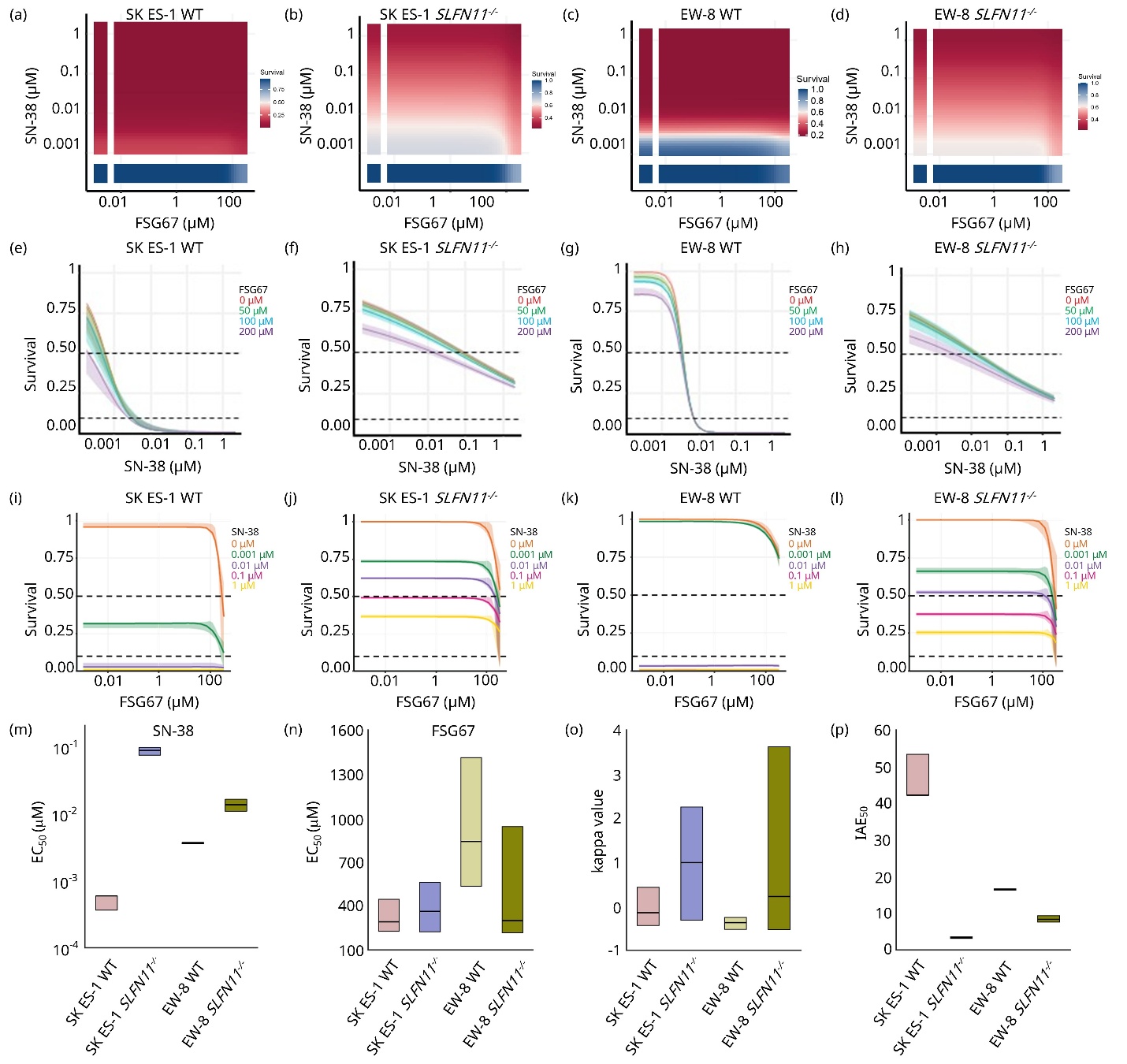
**

**Figure S3. *SLFN11* knockout sensitizes EWS cells to combined DNA damage and *GPAT1* inhibition. (a–i)** BRAID analysis from two pooled bioreplicate experiments testing SK ES‑1 WT and *SLFN11^-/-^* cell line and EW-8 WT and *SLFN11^-/-^* cell line with SN‑38 and FSG67 for 72 h. Response surface plot for SK ES‑1 WT **(a)** SK ES‑1 *SLFN11^-/-^* **(b)** EW‑8 WT **(c)** EW‑8 *SLFN11^-/-^* **(d)** cell lines. **(e-h)** Dose response curve for SN-38 in SK ES‑1 WT **(e)** SK ES‑1 *SLFN11^-/-^* **(f)** EW‑8 WT **(g)** EW‑8 *SLFN11^-/-^* **(h)** cell lines. cells. **(i-l)** Dose response curve for FSG67 in SK ES‑1 WT **(i)** SK ES‑1 *SLFN11^-/-^* **(j)** EW‑8 WT **(k)** EW‑8 *SLFN11^-/-^* **(l)** cell lines. **(m–p)** Quantitative BRAID analysis of the interaction between SN-38 and FSG67 in SK ES‑1 WT and *SLFN11^-/-^* cell line and EW-8 WT and *SLFN11^-/-^* cell line. **(m,n)** Integrated Drug Mean Activity, reported as IDMB for SN-38 **(m)** and IDMA for FSG67 **(n),** representing the BRAID-derived EC_50_ values for the two single agents. **(o)** κ (kappa) values, where values = 0 indicate additivity; < 0 indicate antagonism; 0 indicate synergy. Results were obtained from pooling two independent replicates and are plotted with 95% confidence intervals. **(p)** Integrated Area Excess (IAE), shown as IAE_50_, quantifying the magnitude of combination benefit at 50% inhibition in SK ES-1 WT and *SLFN11^-/-^* and EW-8 WT and *SLFN11^-/-^* cell lines.

**Table S10.** BRAID modeling results for SN-38-based drug combinations in WT and *SLFN11^-/-^* ES-8, SK ES-1, and EW-8 cell lines. IDMA and IDMB represent the EC_50_ values of the partner drug (FSG67) and the anchor drug (SN-38), respectively. The synergy coefficient (κ) quantifies drug interaction, with positive values indicating synergy and negative values indicating antagonism. Near zero values indicate additive effect. κ-lo and κ-hi indicate the lower and upper bounds of the 95% confidence interval for the kappa (κ) interaction coefficient. IAE_50_ measures the combinatorial efficacy required to achieve 50% cell death, rmse is root mean square error.

| **Cell line** | **κ** | **κ-lo** | **κ-hi** | **IDMA** | **IDMB** | **rmse** | **IAE50** |
| --- | --- | --- | --- | --- | --- | --- | --- |
| ES-8 WT | -0.338 | -0.41 | -0.29 | 298 | 0.002 | 0.026 | 22.919 |
| ES-8 *SLFN11^-/-^* | 0.889 | 0.44 | 1.35 | 251 | 0.157 | 0.078 | 2.505 |
| SK ES-1 WT | -0.146 | -0.44 | 0.43 | 293 | 0.001 | 0.035 | 42.164 |
| SK ES-1 S*LFN11^-/-^* | 0.990 | -0.32 | 2.25 | 364 | 0.085 | 0.054 | 3.358 |
| EW-8 WT | -0.370 | -0.52 | -0.25 | 840 | 0.004 | 0.043 | 16.608 |
| EW-8 *SLFN11^-/-^* | 0.221 | -0.53 | 3.62 | 300 | 0.013 | 0.107 | 8.373 |
