# Supplementary material for: Targeting glycerophospholipid biosynthesis overcomes chemoresistance driven by SLFN11 loss in Ewing sarcoma
Source: Cell Death Dis. 2026 Jan 31;17(1):190. doi: 10.1038/s41419-026-08432-7 (PMC12877146; doi:10.1038/s41419-026-08432-7)

**Supplementary Information: Original Western Blots**

**Targeting glycerophospholipid biosynthesis overcomes chemoresistance driven by SLFN11 loss in Ewing sarcoma**

Kasturee Chakraborty^1^, Ritambhar Burman^1^, Saharsh Satheesh^1^, Matthew Kieffer^2^, Chandni Karuhatty^1^, Zuo-Fei Yuan^3^, Haiyan Tan^3^, Ankhbayar Lkhagva^3^, Anthony A High^3^, Xusheng Wang^4,5^, Alaa Refaat^6^, Nathaniel R. Twarog^6^, Weixing Zhang^7^, Yaxu Wang^8^, Yiping Fan^9^, Qian Li^10^, M Madan Babu^8^, Anang A Shelat^6^, Elizabeth Stewart^11^, Michael A Dyer^2^, and Puneet Bagga^1*^

^1^Department of Radiology, St. Jude Children's Research Hospital, Memphis, TN

^2^Department of Developmental Neurobiology, St. Jude Children's Research Hospital, Memphis, TN

^3^Center for Proteomics and Metabolomics, St. Jude Children's Research Hospital, Memphis, TN

^4^Department of Neurology, University of Tennessee Health Science Center, Memphis, TN

^5^Department of Genetics, Genomics and Informatics, University of Tennessee Health Science Center, Memphis, TN

^6^Department of Chemical Biology and Therapeutics, St. Jude Children's Research Hospital, Memphis, TN

^7^Department of Structural Biology, St. Jude Children's Research Hospital, Memphis, TN

^8^Center of Excellence for Data-Driven Discovery, Department of Structural Biology, St. Jude Children's Research Hospital, Memphis, TN

^9^Center for Applied Bioinformatics, St. Jude Children's Research Hospital, Memphis, TN

^10^Department of Biostatistics, St. Jude Children's Research Hospital, Memphis, TN

^11^Department of Oncology, St. Jude Children's Research Hospital, Memphis, TN

Figure 2b: GPD2


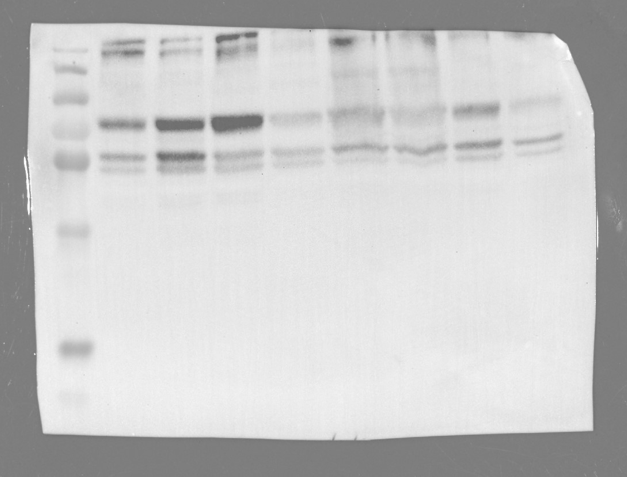


Figure 2b: SLFN11


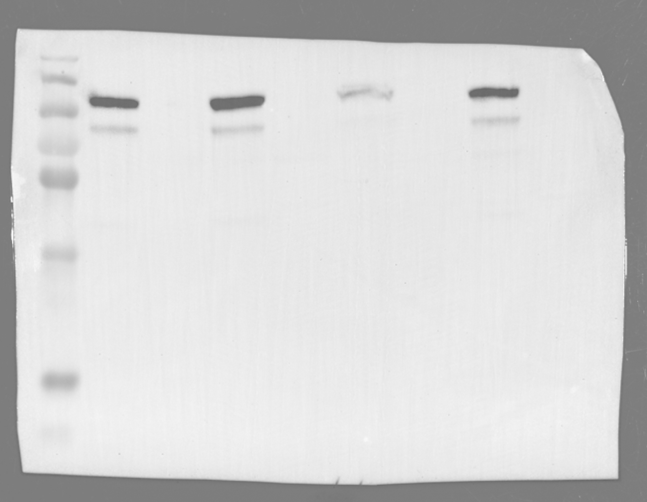


Figure 2b: beta actin


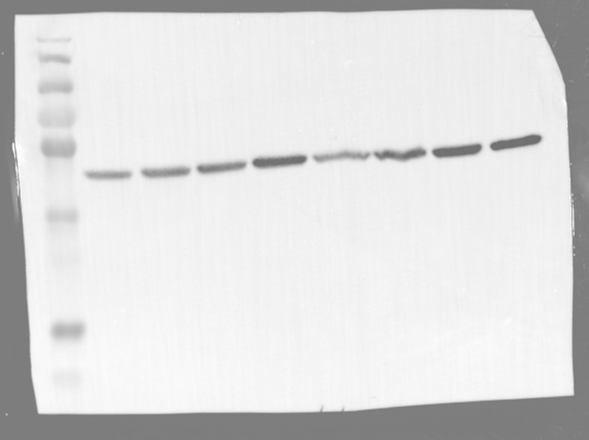

Supplement: Supplementary file 2 — Supplementary Information Original Western Blots [file 41419_2026_8432_MOESM2_ESM.docx]
